# Supplementary material for: Mycobacterium tuberculosis triggers reduced inflammatory cytokine responses and virulence in mice lacking Tax1bp1
Source: PLoS Pathog. 2025 Oct 31;21(10):e1012829. doi: 10.1371/journal.ppat.1012829 (PMC12588459; doi:10.1371/journal.ppat.1012829)
Supplement: S1 Text — (DOCX) [file ppat.1012829.s001.docx]

**Supplemental Figure Legends**

**S1 Fig.** **Tax1bp1-deficiency abrogates *M. tuberculosis* virulence and inflammatory cytokine responses.** (A) In an independent experiment, male and female mice were infected by the aerosol route with *M. tuberculosis*. Male mice were euthanized at 9- and 21- days post-infection*.* Female mice were euthanized at 1- and 50-days post-infection. Lung homogenates were enumerated for CFU. Results are the mean ± SEM from five mice. The mean experimental inoculum was 104 CFU as determined by CFU enumeration at 1-day post-infection. (B) Cytokine levels from infected lung homogenates at 9-, 21-, and 50 days post-infection were measured by ELISA. Results are the mean ± SEM from five samples. The p-values from t-test comparisons are shown.

**S2 Fig.** **Analysis of lung pathology and neutrophil staining in the lungs during *M. tuberculosis* aerosol infection of wild-type and *Tax1bp1^-/-^* mice.** (A) Paraffin-embedded thin sections of lung samples from infected wild-type and *Tax1bp1^-/-^* mice were stained with hematoxylin and eosin (H&E). (B) Pathology was analyzed in H&E-stained images from five infected wild-type and five *Tax1bp1^-/-^* mice at 21- and 50 days post-infection. (C) Paraffin-embedded thin sections of the lung from infected wild-type and *Tax1bp1^-/-^* mice were stained with antibodies against myeloperoxidase. Antibody staining was detected with 3,3'-diaminobenzidine. (D) Quantitative analysis of the percentage of cells that stained positive for myeloperoxidase is shown. Results are the mean ± SEM from five mice. Brackets indicate p-values from t-test comparisons.

**S3 Fig.** **Ubiquitin colocalization with *M. tuberculosis* in the lungs during murine aerosol infection of wild-type and *Tax1bp1^-/-^* mice.** (A) Serial thin sections of paraffin-embedded lung specimens were stained with antibodies against ubiquitin, *M. tuberculosis*, or hematoxylin and eosin. Antibodies were detected with 3,3'-diaminobenzidine. (B) Quantitative analysis of ubiquitin staining pixel overlap with *M. tuberculosis* in overlayed images. Results are mean ± SEM from five samples. The p*-*value from the t-test comparison is shown.

**S4 Fig. Tax1bp1-deficiency limits *L. monocytogenes* growth during murine infection.** In an independent experiment, mice were infected with *L. monocytogenes* by the intravenous route, and CFU were enumerated from spleen and liver homogenates at 48 hours post-infection. Results are the mean ± SEM from five mice. Brackets indicate p-values from t-test comparisons. CFU data were logarithmically transformed prior to statistical analysis.

**S5 Fig. Tax1bp1-deficiency decreases the frequency of microabscess formation and lymphocyte depletion during *L. monocytogenes* infection.** (A, B) Serial thin sections of paraffin-embedded spleen and liver specimens from mice infected by the intraperitoneal route, collected at 72 hours post-infection, were stained with hematoxylin and eosin. (C-E) Pathology was analyzed in H&E-stained images from five infected wild-type and 5 *Tax1bp1^-/-^* mice at 72 hours post-infection.

**S6 Fig. Gating strategy used for the identification of myeloid subsets.** A representative flow panel is shown depicting the gating strategy for the identification and sorting of myeloid subsets. B, T, and NK cells were gated out. AMs (CD11b^lo^CD11c^hi^SiglecF^hi^), MNC1 (SiglecF^-^CD11b^+^CD11c^lo^MHCII^+^), MNC2 (SiglecF^-^CD11b^+^CD11c^hi^MHCII^hi^), and neutrophils (Neut; SiglecF^-^Ly6G^hi^CD11b^hi^) were sorted. The gating strategy used to identify ZsGreen-positive cells is shown in the bottom row.

**S7 Fig. Gating strategy used for the identification of NK, T, and B cells.** A representative flow panel is shown depicting the gating strategy for the identification and sorting of the following immune cell subsets: NK cells (CD3^-^NK^+^), B cells (CD3^-^CD19^+^), CD4^+^ T cells (CD3^+^CD4^+^), and CD8^+^ T cells (CD3^+^CD8^+^). The gating strategy used to identify ZsGreen-positive cells is shown in the bottom row.

**S8 Fig. Tax1bp1-deficiency reduced *Mtb* growth in AMs and MNC2.** Mice were infected with aerosolized *Mtb* expressing ZsGreen (calculated dose of 100 CFU per mouse). Five female wild-type and five *Tax1bp1^-/-^* mice were euthanized at 7- and 14-days post-infection, and five male mice of each genotype were euthanized at 21-days post-infection. (A) Lung and spleen homogenates from 5 wild-type and 5 *Tax1bp1^-/-^* mice at each time point were plated for CFU. (B) Lung cells were pooled and stained for AMs, neutrophils, and recruited MNC1s and MNC2s. Innate immune cells were sorted. The sorted cells were plated for *Mtb* CFU in quadruplicate. Data were normalized to the number of cells sorted. (C) ZsGreen-positive innate immune cell subsets were quantified by analytical flow cytometry (experiment 1). An independent second biological replicate infection was performed in pooled lung cells from *Mtb-*infected female mice (experiment 2). Finally, the mean Zs-Green counts from the data shown in Fig. 3D are displayed (experiment 3). SEM and p values from the t-test are displayed.

**S9 Fig. Tax1bp1-deficiency restricts *Mtb* growth in sorted AMs.** BAL cells from wild-type (A) and *Tax1bp1^-/-^* (B) mice were stained with a live/dead stain and antibodies for SiglecF and CD11c. Live CD11c+SiglecF+ AMs were sorted and infected with luciferase-expressing *Mtb* at a M.O.I. of 1. (C) Luminescence measurements of *Mtb-*infected monolayers were obtained daily and graphed as the fold change in luminescence relative to day 0, immediately post-infection. At 3 days post-infection, monolayers were lysed for CFU enumeration. The p-values from t-test comparisons are shown.

**S10 Fig. Tax1bp1-deficiency reduces autophagy flux.** Macrophages were (A) unstimulated or (B) subjected to nutrient deprivation by incubation with Earle’s Balanced Salt solution (EBSS) followed by treatment with bafilomycin. Cell lysates were analyzed by SDS-PAGE and immunoblot with antibodies to LC3 and actin. The ratios of the LC3-II:LC3-I band densities are displayed. Data are representative of two independent experiments.

**S11 Fig. Tax1bp1-deficiency abrogates the colocalization of *Mtb* with LC3.** AMs were infected *ex vivo* with ZsGreen-expressing *Mtb* Erdman at a M.O.I of 2. At 8- and 24-hours post-infection, monolayers were fixed and stained with primary antibodies for autophagy markers, secondary Alexa-Fluor 647 antibodies, and DAPI. Immunofluorescence microscopy was performed at 63X magnification in 69 x/y positions and 4 z planes each in quadruplicate wells. Immunofluorescence microscopy images at 8- (A) and 24- (B) hours post-infection are displayed. Arrows denote *Mtb* that colocalized with LC3 or ubiquitin. The white bar denotes 10 µm. (C) Quantification of *Mtb* and autophagy marker colocalization is displayed. Mean percent colocalization in each well, SEM, and p-values from the t-test are depicted. Data are representative of two independent experiments.

**S12 Fig. Principal component analysis (PCA) of changes in global protein abundance during *Mtb*- or mock-infection of AMs.** In five independent biological replicate experiments, AMs were mock- or *Mtb-*infected. AMs were harvested at 24 hours post-infection for global protein abundance analysis. The *Mtb-*infected samples are labeled TB.

**S13 Fig. Pathogen and host differential gene expression analysis volcano plots.** Volcano plots display the differentially regulated genes from (A) *Mtb* and (B) the host during wild-type and *Tax1bp1^-/-^* AM infection with *Mtb*. The volcano plots display the log_2_fold change of normalized mean hit counts in wild-type vs. *Tax1bp1^-/-^* samples and -log_10_(adj. p-value for host genes or unadjusted p*-*value for *Mtb* genes). Colors denote genes that were upregulated (purple) or downregulated (green) in wild-type compared to *Tax1bp1^-/-^* samples.

**S14 Fig. Tax1bp1-deficiency reduces necrotic-like cell death and accelerates apoptosis during *Mtb* infection of AMs.** As described in the Figure 8 legend, AMs were infected with *Mtb* at a M.O.I. of 1 in the presence of PI (propidium iodide) and CellEvent without (A) or with (B) IFN-γ added to the media. Fluorescence images were obtained at 20X magnification in two positions per well in three replicate wells. Representative fluorescence and brightfield microscopy images are displayed. The white bar is 100 µm. Data are representative of three independent experiments.

**S15 Fig. Tax1bp1-deficiency accelerates apoptosis during *Mtb* infection of IFN-γ-stimulated BMDMs.** BMDMs were infected with *Mtb* at a M.O.I. of 1 in the presence of PI (propidium iodide) and CellEvent without (A, C) or with (B, D) IFN-γ added to the media. Fluorescence images were obtained at 20X magnification in two positions per well in three replicate wells. Representative fluorescence and brightfield microscopy images are displayed. The white bar denotes 100 µm. (C, D) The number of fluorescent cells in each field was quantified in the green (CellEvent) and red fluorescence (PI) channels. Fluorescent cell numbers were normalized by the total number of cells in the brightfield image for each field. Mean, SEM, and statistically significant FDR-adjusted p-values comparisons are displayed. For clarity, only statistically significant p-values (p < 0.05) are displayed. Data are representative of two independent experiments.

**S16 Fig. Tax1bp1-deficiency induces apoptosis in uninfected IFN-γ-stimulated AMs.** Uninfected macrophages were incubated in the presence of PI (propidium iodide) and CellEvent without (A, B) or with (B, C) IFN-γ added to the media. Fluorescence images were obtained at 20X magnification in two positions per well in three replicate wells. Representative fluorescence and brightfield microscopy images are displayed. The white bar denotes 100 µm. (B, D) The number of fluorescent cells in each field was quantified in the green (CellEvent) and red fluorescence (PI) channels. Fluorescent cell numbers were normalized by the total number of cells in the brightfield image for each field. Mean, SEM, and statistically significant FDR-adjusted p-values comparisons are displayed. For clarity, only statistically significant p-values (p < 0.05) are displayed. Data are representative of two independent experiments.
