# Supplementary figures and images for: Mycobacterium tuberculosis triggers reduced inflammatory cytokine responses and virulence in mice lacking Tax1bp1
Source: PLoS Pathog. 2025 Oct 31;21(10):e1012829. doi: 10.1371/journal.ppat.1012829 (PMC12588459; doi:10.1371/journal.ppat.1012829)

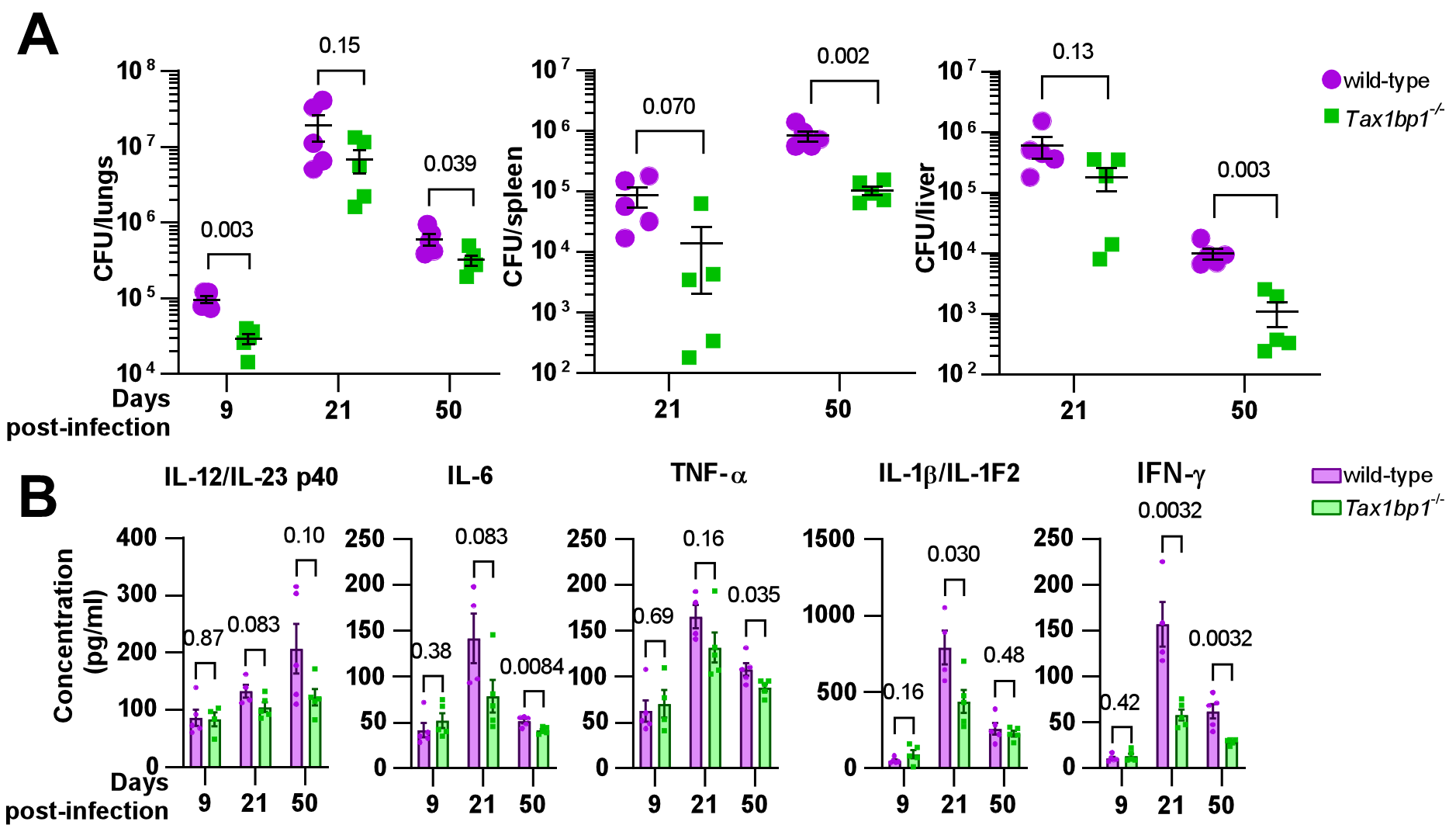

Supplement: S1 Fig — (TIF) [file ppat.1012829.s002.tif]

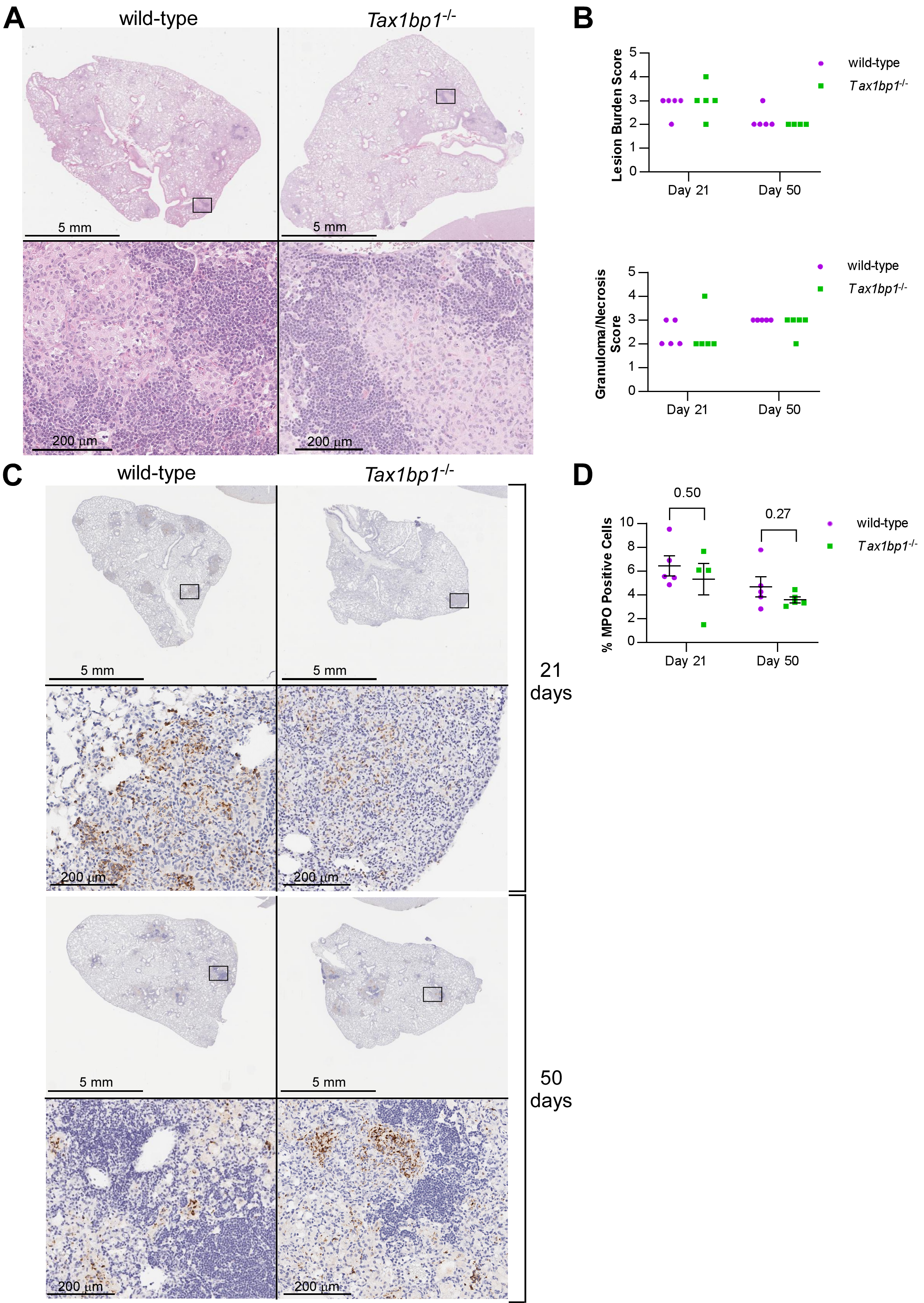

Supplement: S2 Fig — (TIF) [file ppat.1012829.s003.tif]

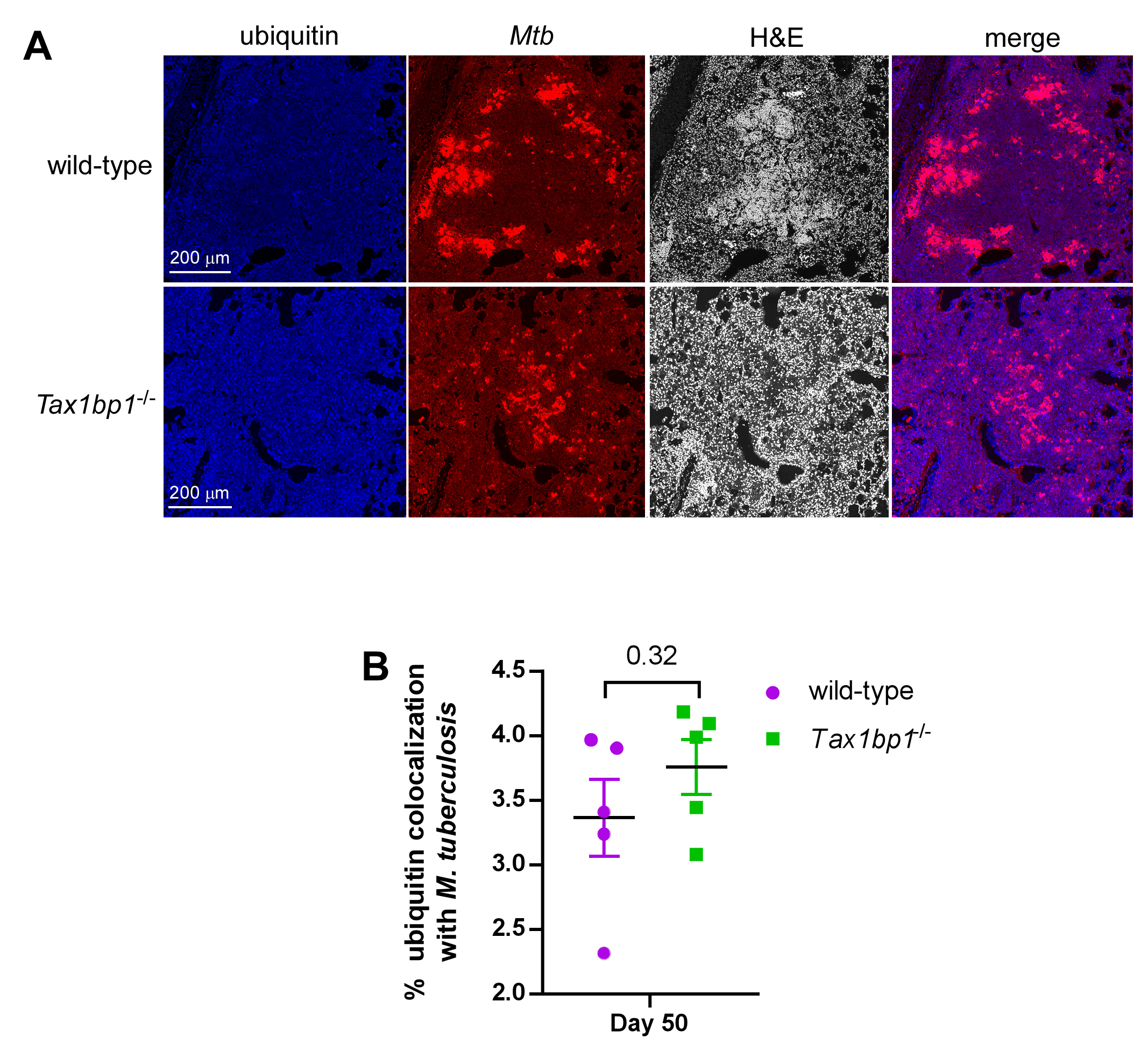

Supplement: S3 Fig — (TIF) [file ppat.1012829.s004.tif]

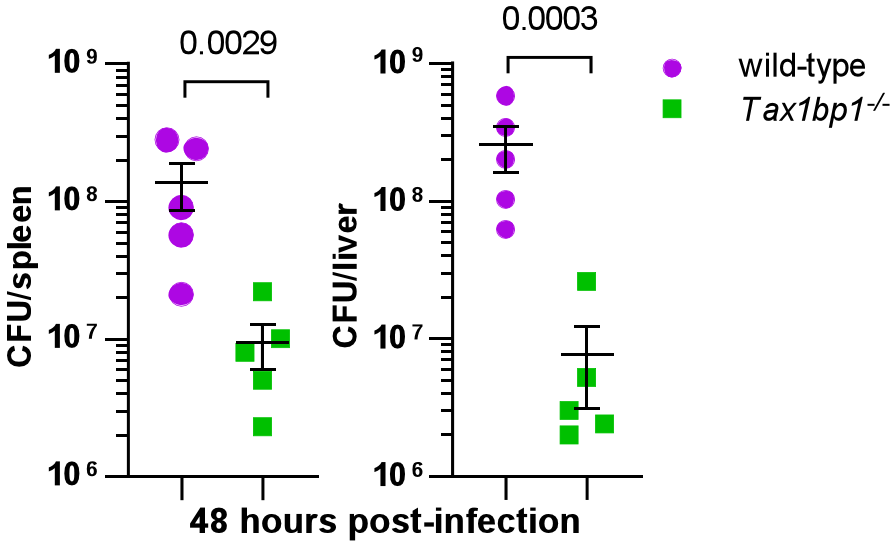

Supplement: S4 Fig — (TIF) [file ppat.1012829.s005.tif]

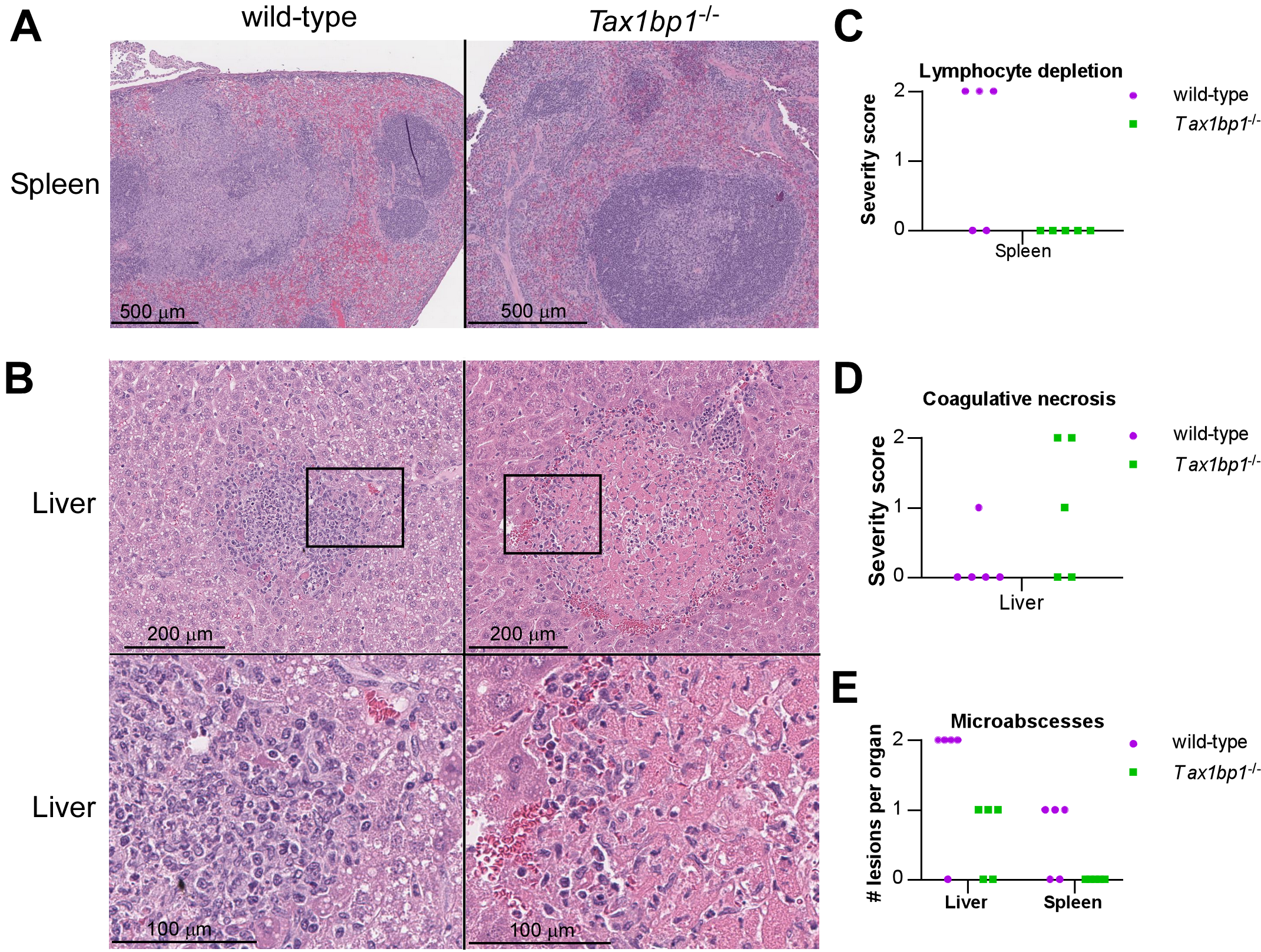

Supplement: S5 Fig — (TIF) [file ppat.1012829.s006.tif]

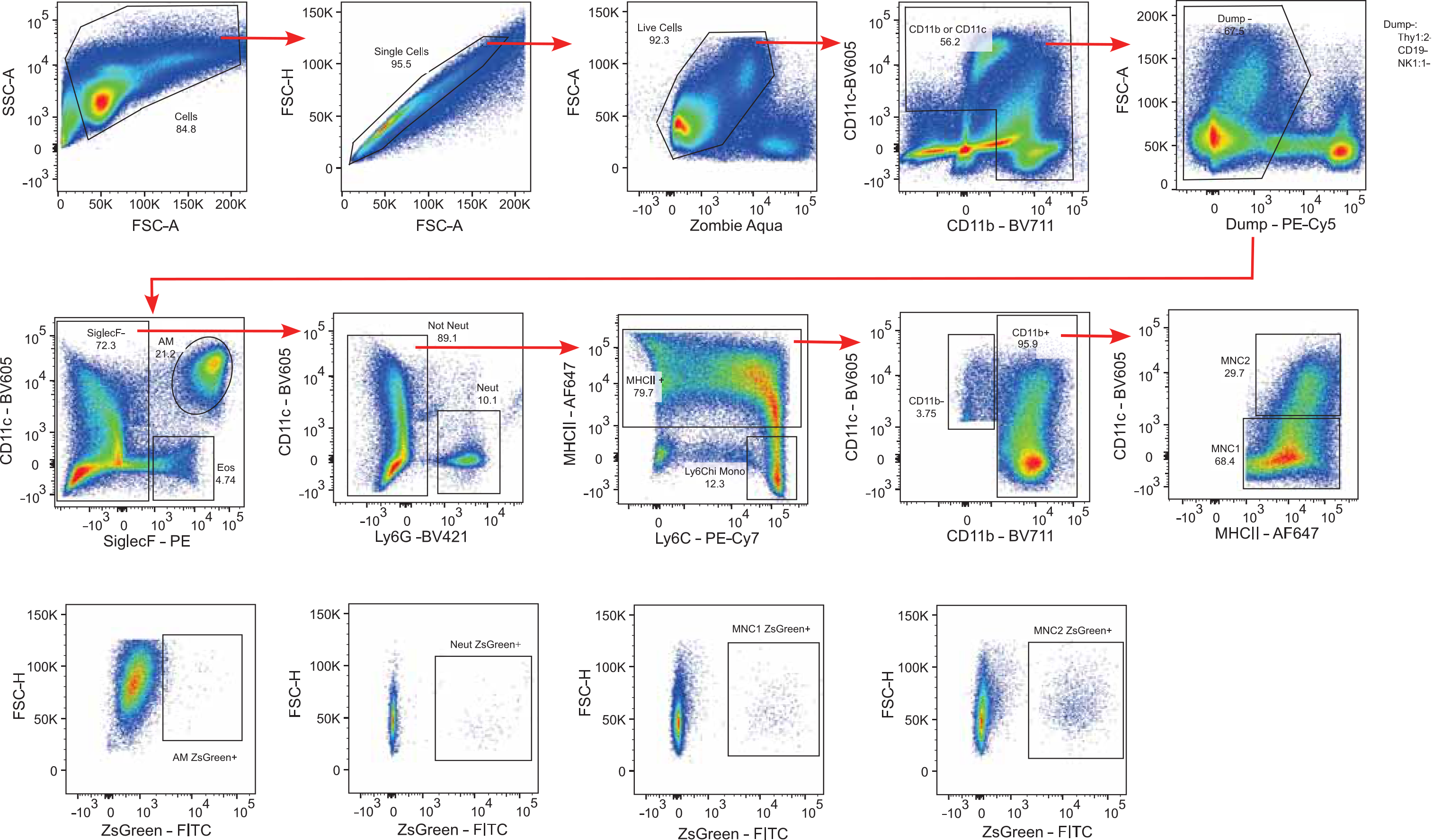

Supplement: S6 Fig — (TIF) [file ppat.1012829.s007.tif]

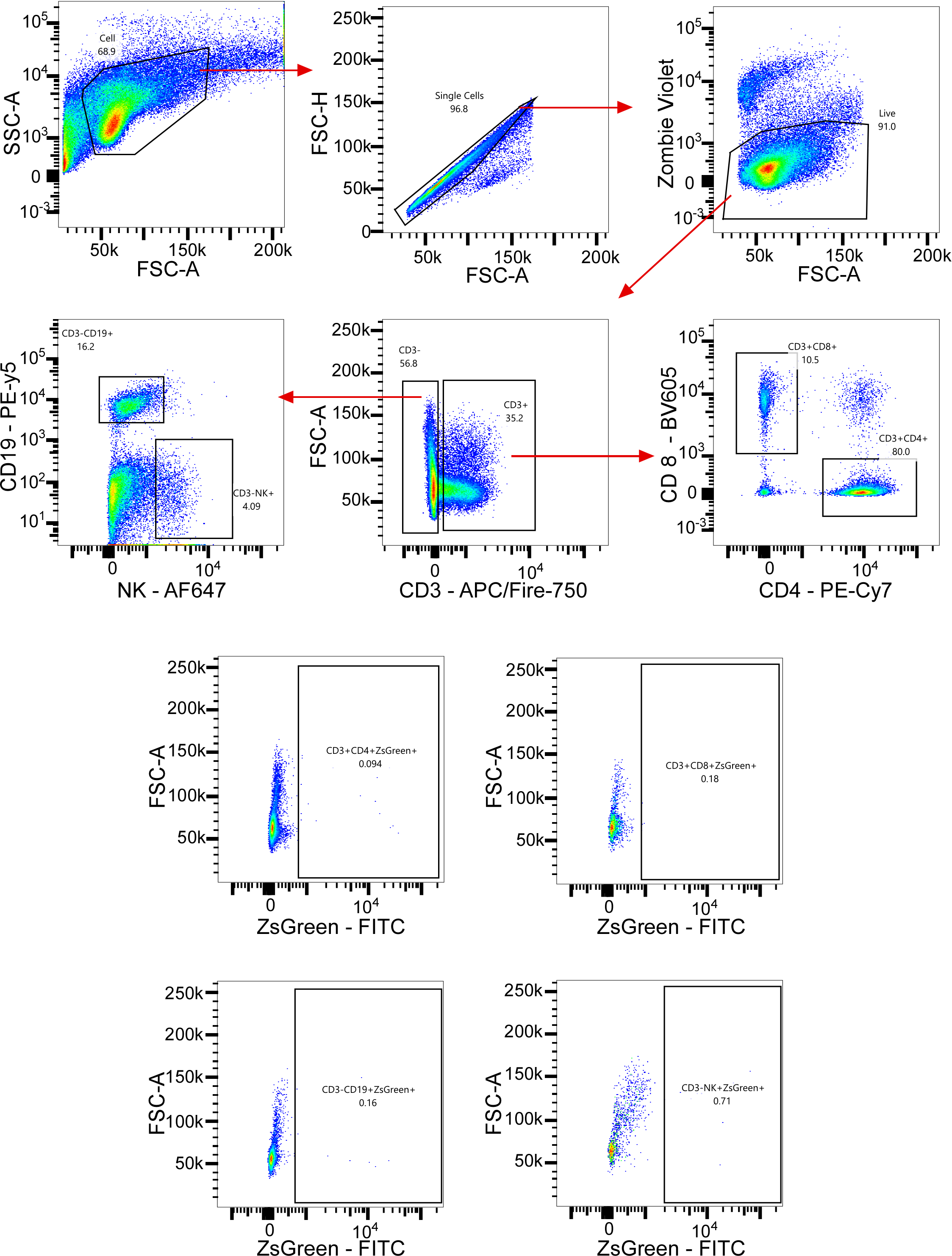

Supplement: S7 Fig — (TIF) [file ppat.1012829.s008.tif]

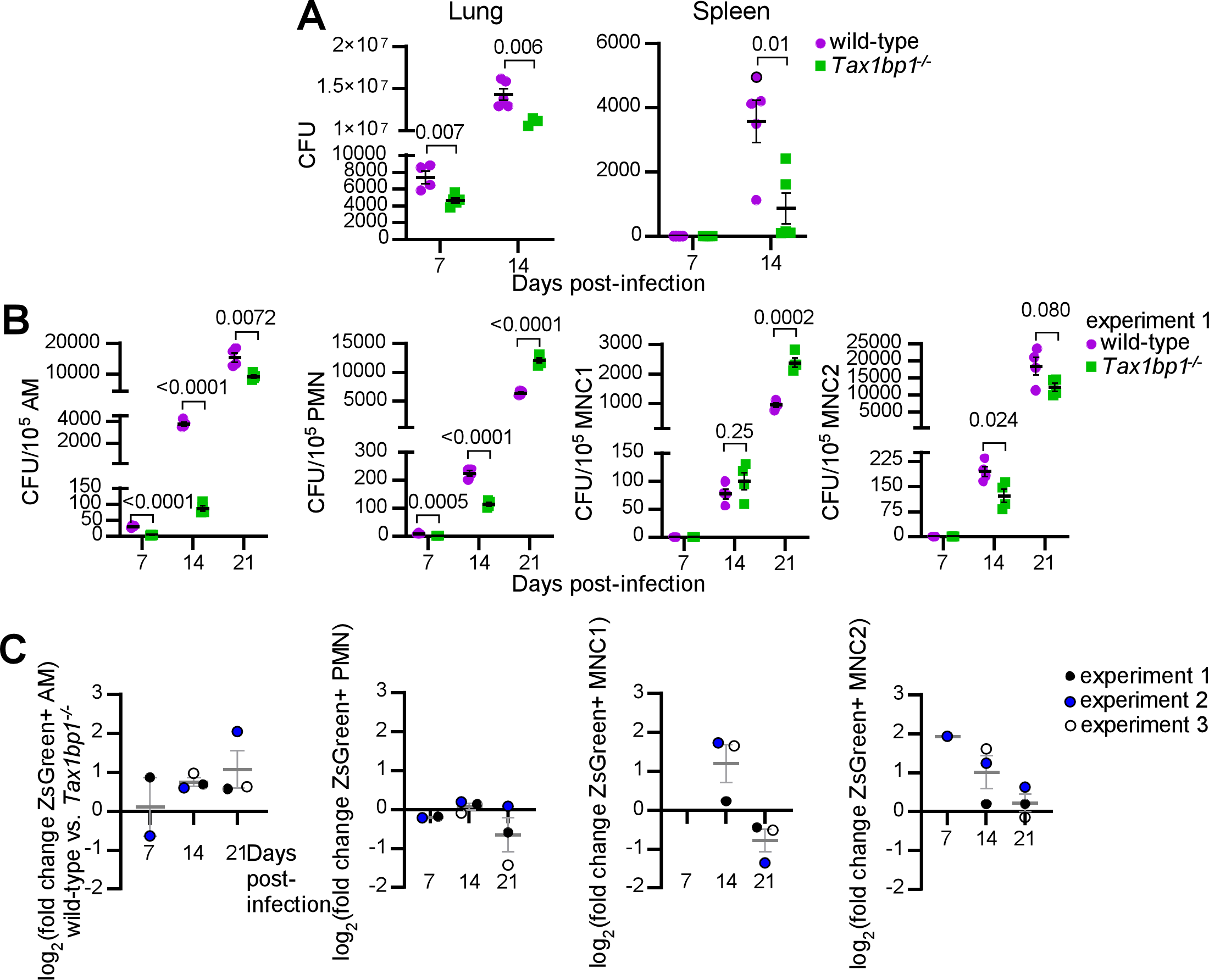

Supplement: S8 Fig — (TIF) [file ppat.1012829.s009.tif]

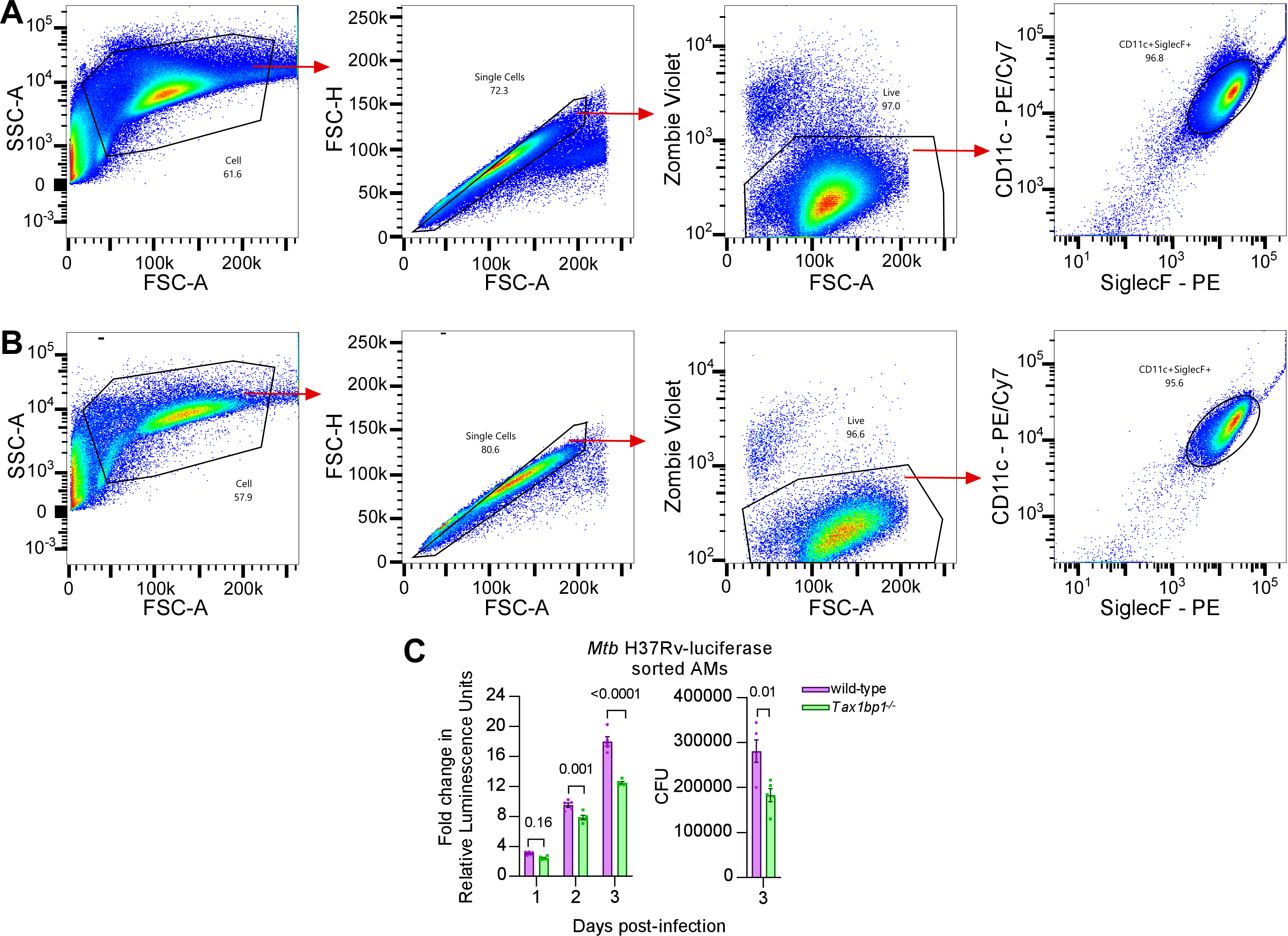

Supplement: S9 Fig — (TIF) [file ppat.1012829.s010.tif]

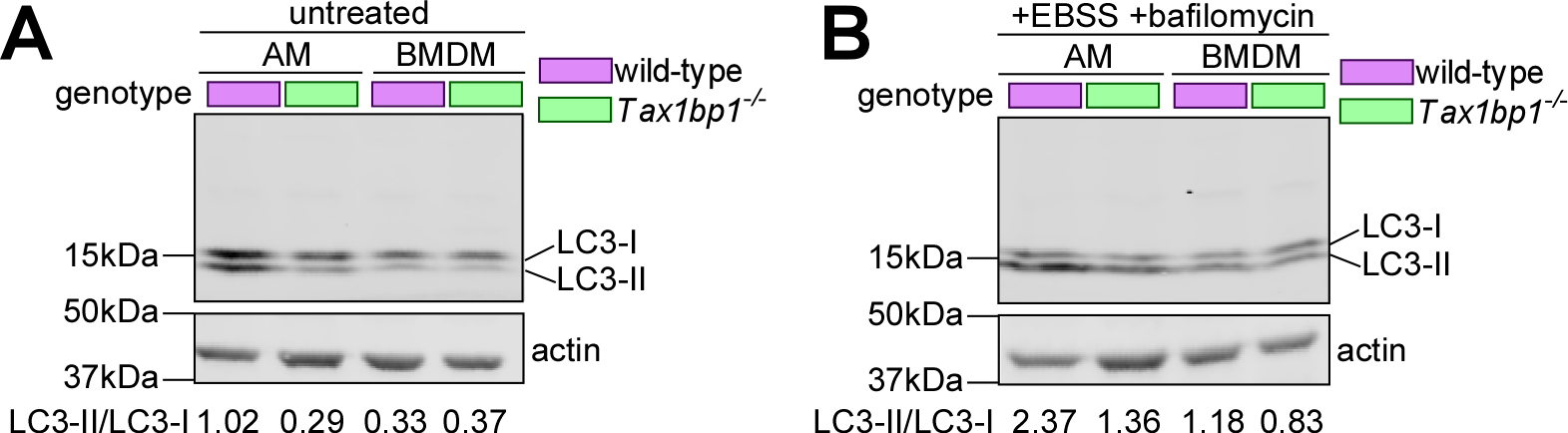

Supplement: S10 Fig — (TIF) [file ppat.1012829.s011.tif]

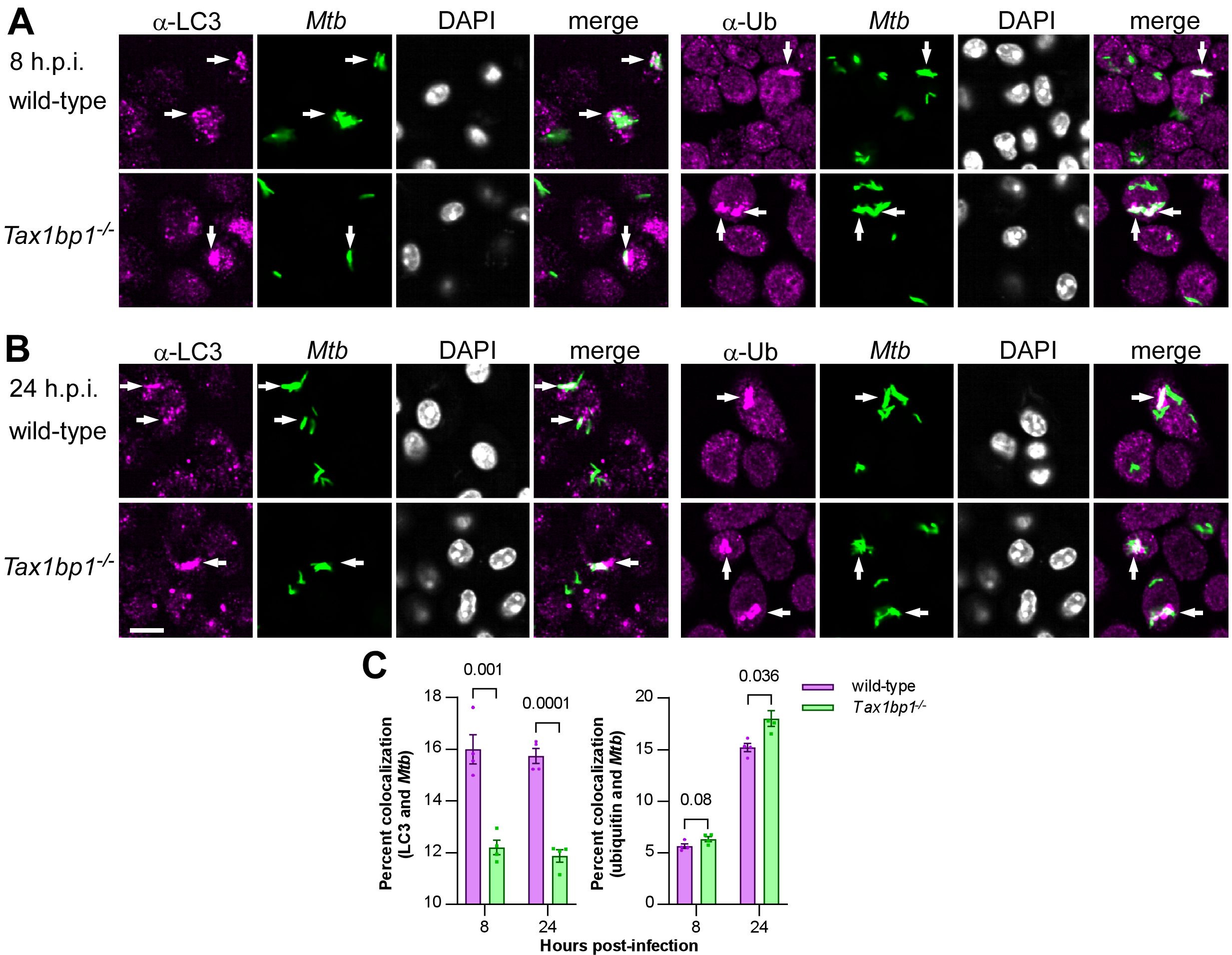

Supplement: S11 Fig — (TIF) [file ppat.1012829.s012.tif]

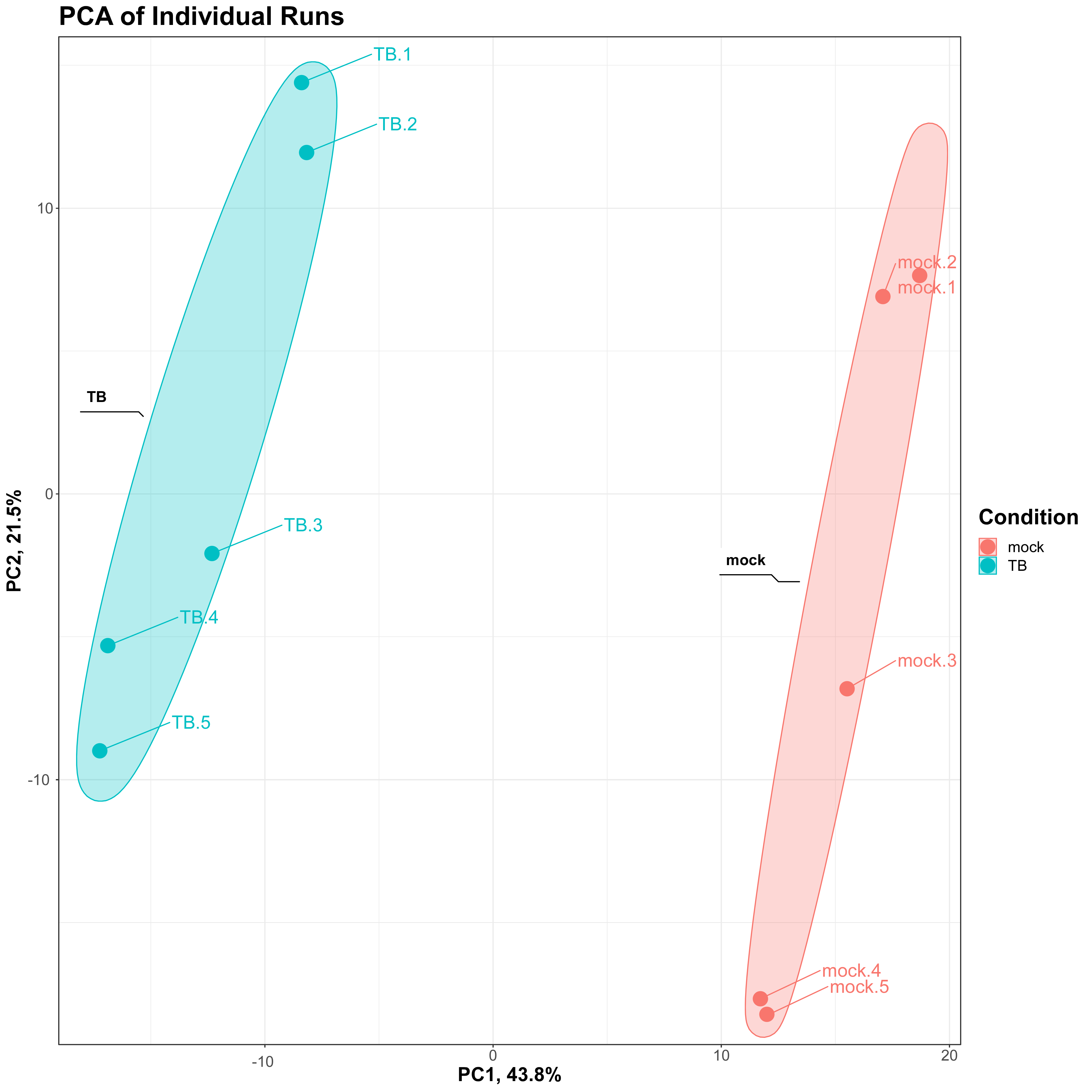

Supplement: S12 Fig — (TIF) [file ppat.1012829.s013.tif]

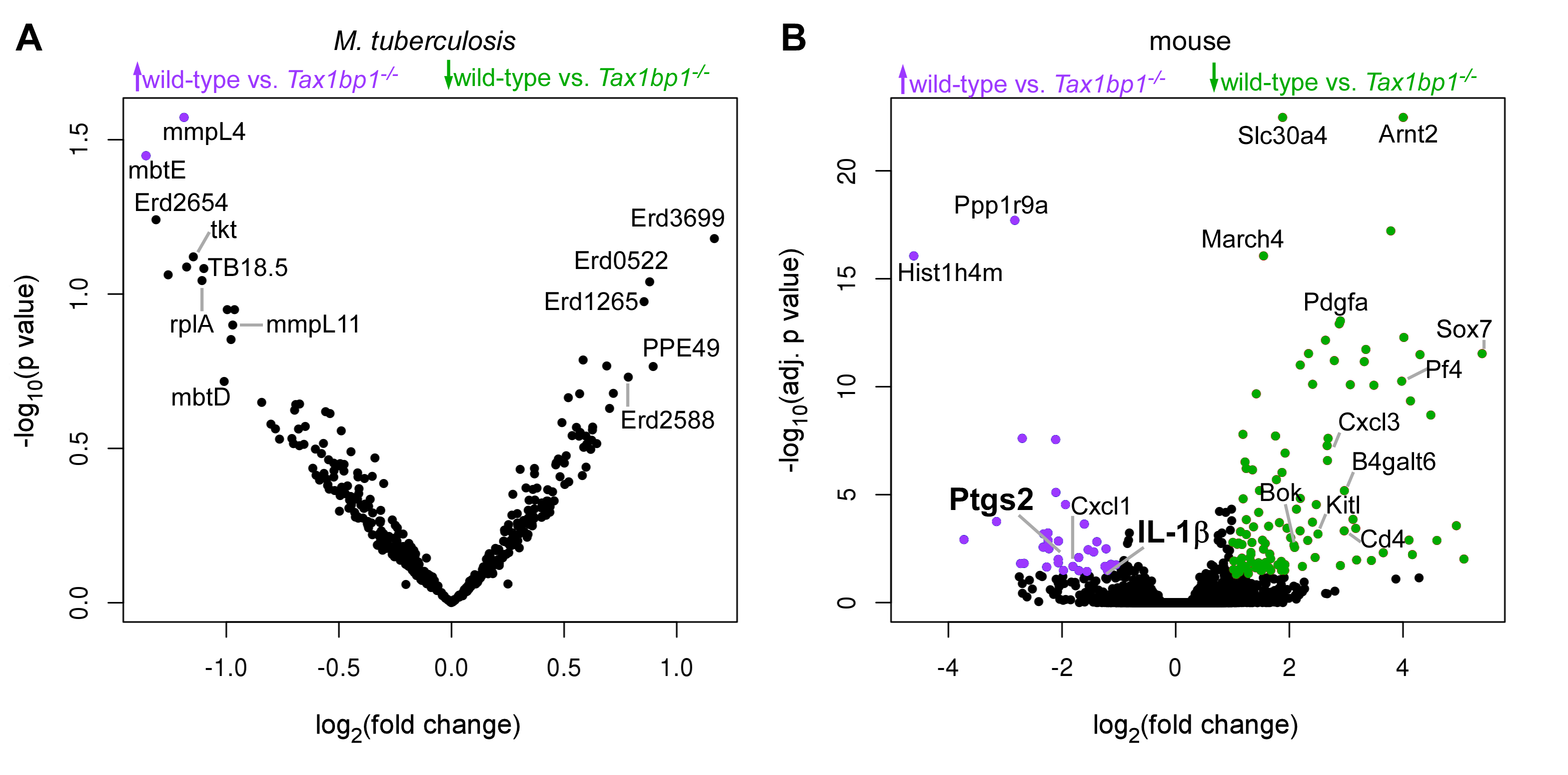

Supplement: S13 Fig — (TIF) [file ppat.1012829.s014.tif]

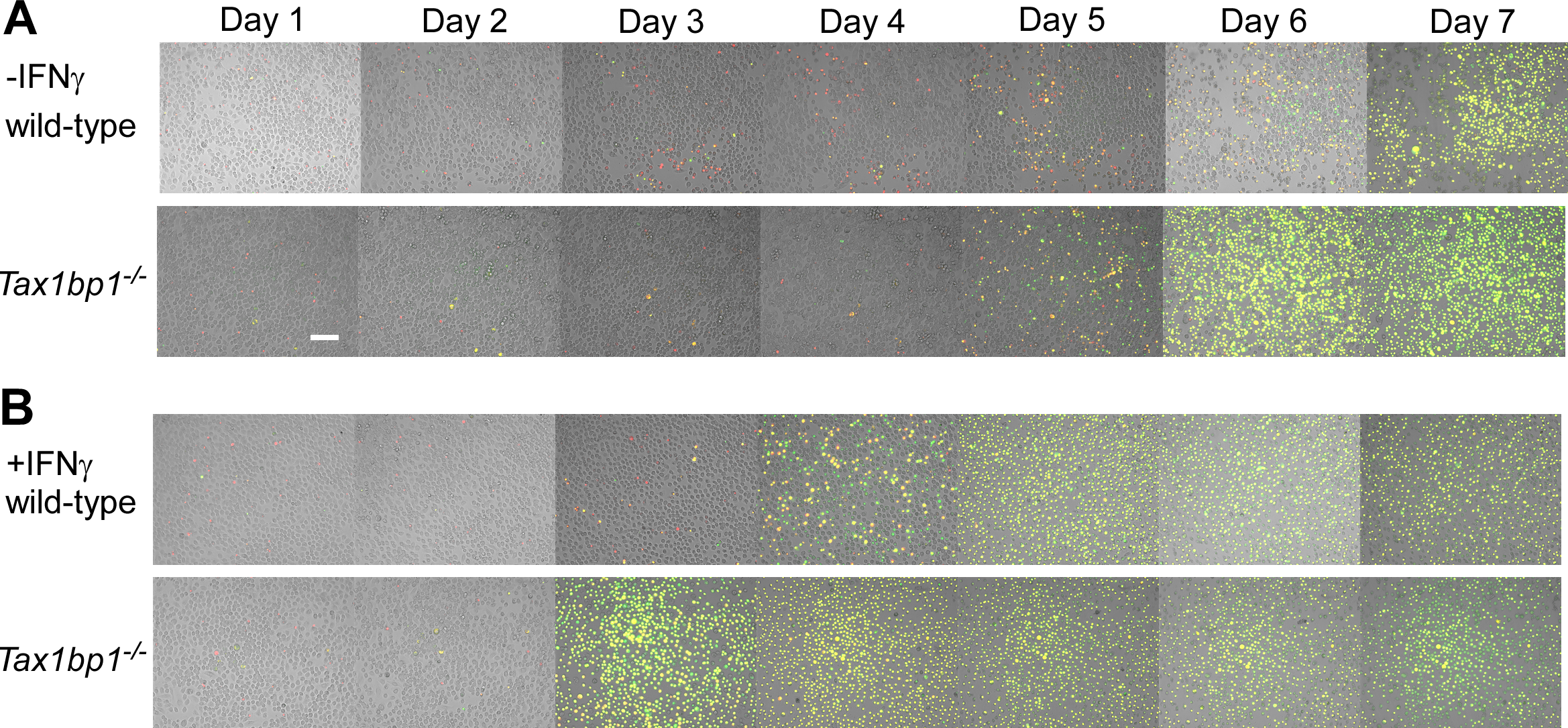

Supplement: S14 Fig — (TIF) [file ppat.1012829.s015.tif]

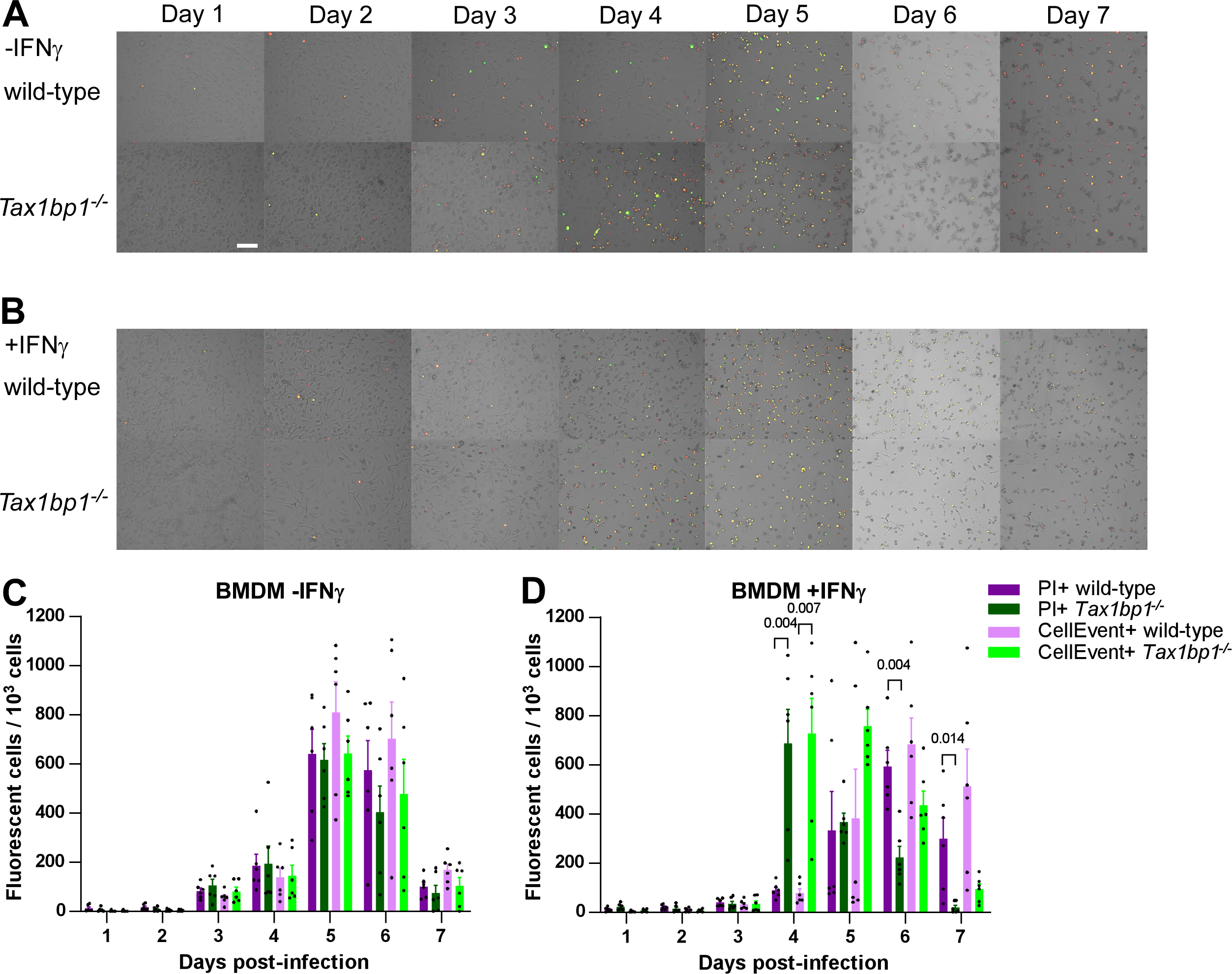

Supplement: S15 Fig — (TIF) [file ppat.1012829.s016.tif]

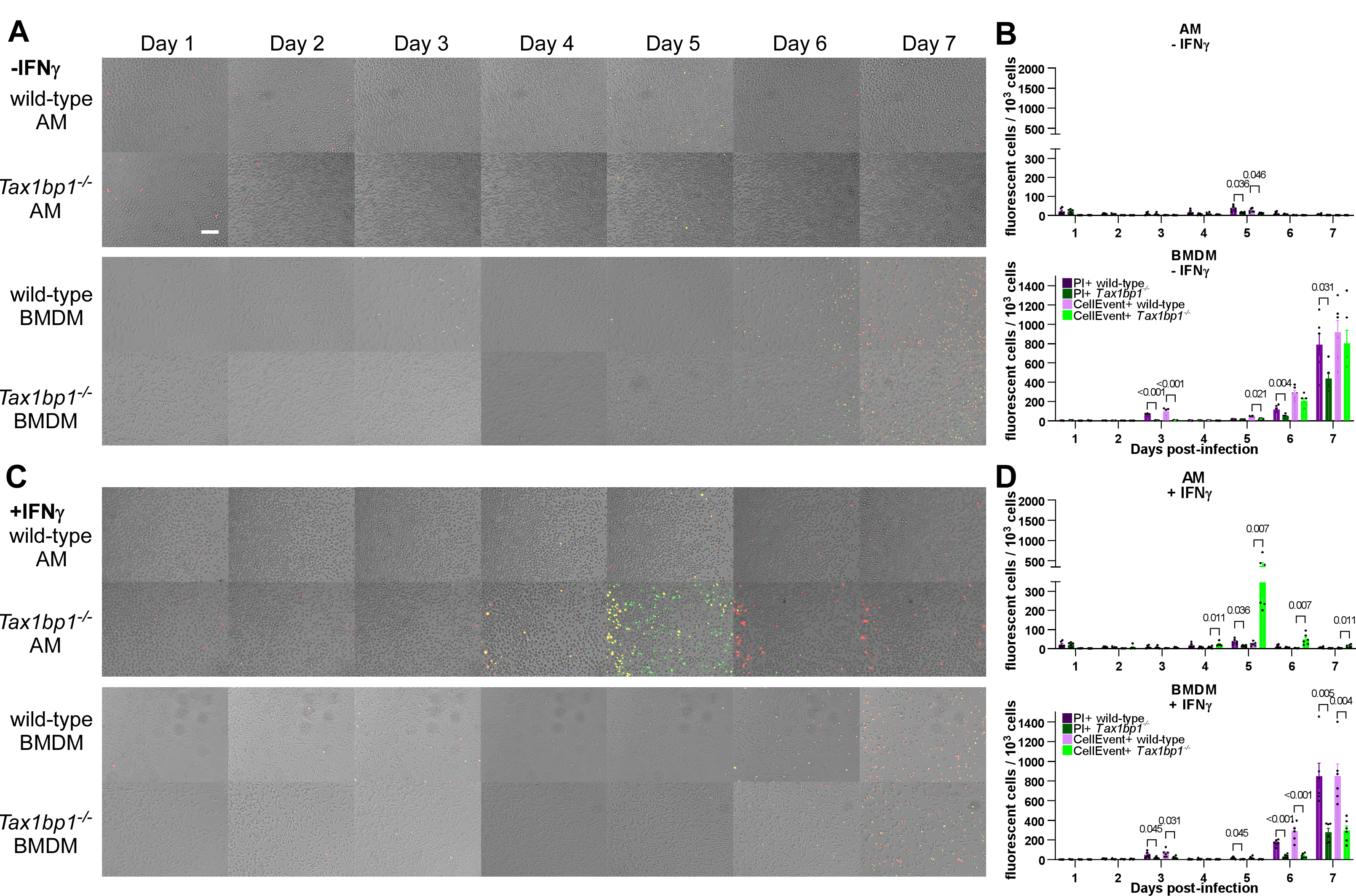

Supplement: S16 Fig — (TIF) [file ppat.1012829.s017.tif]
