# Supplementary material for: Mycobacterium tuberculosis triggers reduced inflammatory cytokine responses and virulence in mice lacking Tax1bp1
Source: PLoS Pathog. 2025 Oct 31;21(10):e1012829. doi: 10.1371/journal.ppat.1012829 (PMC12588459; doi:10.1371/journal.ppat.1012829)
Supplement: Attachment — Submitted filename: 20250918 Response to Reviewers #2.pdf [file ppat.1012829.s020.pdf]

UCSF
University of California
San Francisco

Jonathan Budzik, M.D., PhD
Assistant Professor
Department of Medicine
University of California, San Francisco
Address:
513 Parnassus Ave.
Health Sciences East
Room 409
San Francisco, CA 94143

September 22, 2025

Dear Reviewers:

Thank you for considering our research article entitled "Tax1bp1 enhances bacterial virulence and promotes inflammatory responses during Mycobacterium tuberculosis infection" for publication in PLoS Pathogens. We are grateful for the suggestions for improvement. To provide a timely revision of the manuscript, we prioritized our experiments as highlighted in the point-by-point response to the reviewers' comments and concerns.

REVIEWER 3 COMMENTS :

(1) A major issue of the revised manuscript relies on the lack of statistical analysis across different biological replicates and experiments. Importantly, for the Mtb infection analysis in different cell types, it is unclear why the authors decided to represent 3-4 independent experiments in different figures. Also, the infection times do not match. Not only it is hard to read, but it also prevents any robust validation of the findings regarding TAX1BP1 effect across several cell types, which is an important message of the paper.
Specifically, for the Zeon Green analysis, the individual experiments are shown in Figure 3, Figure 3-supp1, Figure 3-suppl2 and Figure 1-supp5. For CFU analysis, the individual experiments are spread in Figure 3, Figure 3-supp2 and Figure1-supp5. I believe this is not good practice.

Good point. Previously, Fig1-supp5 displayed the ZsGreen+ counts from individual mice, whereas the other figures displayed data from pooled mouse samples. We had to pool lung samples from individual mice to have enough material for CFU analysis.

In the revised manuscript, Fig1-supp5 has been relabeled as Fig. 3. The data obtained from three independent animal infection experiments have been consolidated to enable statistical analysis across the different independent experiments.

The data previously displayed in Fig. 3, Fig. 3-supp1, and Fig. 3-supp2 have been consolidated into one Fig. 3-S3C. Fig. 3-S3C displays the mean ZsGreen+ *Mtb* counts displayed in Fig. 3D (experiment 3) as well as the ZsGreen+ counts from two other independent animal infection experiments with pooled samples (experiment 1-2). This analysis demonstrated a statistically significant cell type-specific impact of Tax1bp1-deficiency on the mean *Mtb* Zs-Green+ counts across three independent biological replicate experiments.

REVIEWER 4 COMMENTS:

(1) But the manuscript reads like a few small loosely related phenotypes pieced together. It has distractive in vivo studies and listeria data deviating from the main story.

To better link the different components of the study, and clarify the goal of the experiments involving Listeria, we have reorganized the manuscript to present our findings with *Listeria* immediately following the similar findings with *Mtb.* This helps the reader appreciate that Tax1bp1-deficiency abrogated CFU and systemic cytokine responses even during infection with a pathogen that evades selective autophagy (*Listeria*). (See comment (5) below as well.) Thus, likely other mechanisms explain the reduction in bacterial growth in Tax1bp1-deficient mice.

(2) The manuscript also suffers from an unclear definition of "inflammatory response". Which of inflammatory responses is tax1bp1 regulating? Is it cell death? Is it AM production of IL1b, IFNg or PGE2? Or immune response after Mtb infection? Each of these "inflammatory responses" has a completely distinct meaning.

We agree with this comment. Therefore, we changed the title to specific inflammatory cytokine responses. In a revised model (Fig. 8E), we show that Tax1bp1-deficiency regulates necrotic-like cell death and inflammatory mediators (IL-6, IL1-β, and PGE_2) production.

(3) 1. I do not understand why CFU results at 50 dpi are presented in Figure 1B when the mice appear to have succumbed to the infection much later, not until after 30 weeks, and the role of tax1bp1 in survival doesn't appear even later. This discoordination between CFU/cell death/mortality suggest that the defect in controlling bacterial replication does not contribute directly to host mortality. This needs to be carefully discussed.

We tried to clearly distinguish phenomena in the (i) early and (ii) late stages of infection in the previous submission. In this particular case, we presented CFU results at 50 dpi to show that Tax1bp1 plays an important role in controlling *Mtb* growth when the adaptive immune response is fully activated from T cells.

In the revised manuscript, we've added the following sentence to the discussion: 'Indeed, the possible effects of Tax1bp1-deficiency on other cell types may play an important role in contributing to host mortality during later stages of infection and disease'. (page 20, line 518)

We also wish to point out that C57Bl/6 mice are relatively resistant to mycobacterial infection compared to other mouse strains (see https://doi.org/10.1016/j.vaccine.2009.06.110, and DOI: 10.1046/j.1365-2567.1998.00419.x). Furthermore, others have demonstrated that bacterial CFU differences precede survival differences during murine infection with different *Mtb* strains (doi: 10.1073/pnas.091096998).

(4) Figure 1D. Tax1bp1 deficient mice has lower IFNg and other cytokines in the infected lungs. For the record, IFNg is produced by T cells. Yet the authors found increased CD8 T cell levels. How does decreased IFNg reconcile with increased T cells?

There may be a misunderstanding here, as *we observed decreased levels of CD8 T cells in mice lacking Tax1bp1*; see Fig. 3-S4.

(5) Figure 2E is poorly labeled, please include time point in the figures. For this whole figure, I don't understand the role of Listeria data. The only common thing between listeria and Mtb might be that, after Listeria infection there is a reduced production of multiple cytokines, several of which involved in controlling Listeria. But this has nothing to do with AM or may not have to do with any type of macrophages. what's the point of including Listeria? The authors stated this part is to "confirming the importance of our findings with another intracellular pathogen". what findings are they referring to?

In response to this comment, the label for Fig. 2E has been adjusted. We edited the text to further explain the rationale for experiments using, and importance of the findings in, Listeria-infected mice. Importantly, cytokine production and bacterial growth decreased in Tax1bp1-deficient mice with both *Mtb* and *Listeria*.

(6) In Figure 3-S2B, tax1bp1 deficiency results in decreased bacterial burden in AM, but increased CFU in PMN and MNC1, at 21 dpi. Why?

The underlying mechanism driving the cell type-specific growth of *Mtb* in AMs vs. PMN and MNC1 is still under investigation. In this manuscript, we followed up on this discovery by studying phenotypes in AMs and BMDMs *ex vivo* because *in vivo* mechanistic studies are much more challenging.

(7) Since the CFU results obtained from PMN and MNC1 is different between before and after 21dpi, why is the overall lung burden shows similar reduction in Tax1bp1 deficient mice at 7, 11, 14, 21, and 50 dpi?

This can be explained by the observation that the reduction in CFU (and ZsGreen+ counts) in Tax1bp1-deficient AMs became more pronounced on day 21 and the fact that the absolute number of *Mtb* CFU in MNC1 is relatively low compared to the other cell types; see Fig. 3C. Thus, even though the CFU in the PMN and MNC1 are indeed increased in Tax1bp1-deficient cells on day 21, most of the live CFU were in AMs and MNC2, which exhibited decreased *Mtb* growth in the Tax1bp1-deficient cells. To make this point clearer, alongside the CFU data (Fig. 3B), I have replotted the ZsGreen+ *Mtb* counts, including the data from three independent biological replicate experiments (Fig. 3C).

(8) Figure 5 and 6. The whole section on RNA-seq leads to no meaningful conclusion. I have the same question as one other reviewer: intrinsic apoptotic pathway is the most upregulated in WT AMs. Adding to the confusion, they also state that pro-apoptotic genes are among the top upregulated genes in KO AMs. This contradiction undermines their conclusion that Tax1bp1 differentially regulates necrotic and apoptotic pathways. Adding on that, if, as the authors stated in response letter, complexity of signalling pathways limits the predicting power of RNA-seq data, why having it in the manuscript?

For clarification, the gene ontogeny enrichment analysis (Fig. 6) was performed on the differentially expressed genes up- and down-regulated in *Tax1bp1*$^{-/-}$ AMs compared to wild-type AMs. We edited the figure legend to emphasize this point (page 23, line 602). We also included in the figure itself that the genes analysed in the gene ontogeny enrichment analysis exhibited a log2(fold change) **>1 or <-1** and an adjusted p value < 0.05. The differentially regulated genes in the apoptotic signalling pathway were upregulated in *Tax1bp1*$^{-/-}$ AMs (page 15, lines 389-390), more so than the wild-type AMs (lines 390-391).

RNA-seq was an unbiased approach that led us to generate hypotheses about which functional pathways might be differentially regulated in wild-type vs. Tax1bp1-deficient AMs. The transcriptional profiling led us to further

investigate cell death, inflammatory cytokine, and specifically prostaglandin E2 responses during *Mtb* infection of AMs. We made additional edits to the text that highlight this point (pages 14-15, lines 361-366).

(9) in Figure 7, what are the authors trying to do with cytokine secretion from AMs? Are the authors trying to link observation in Figure 1, IL1b level differs at 11dpi from Mtb infected mice? However, IL1b is higher in tax1bp1 deficient mice, contradicting to figure 7. If the authors believe tax1bp1 regulation of PGE2 is the cause, addition experiment is needed to solidify this point.

Thank you for sharing this observation and comment. The p-value for the IL1b at 11dpi in Figure 1 was incorrect; it should have been 0.10 rather than 0.01. This error has been corrected.

In Fig. 7, we show that Tax1bp1-deficiency reduced cytokine secretion during *Mtb* infection of AMs, which is different from our previously published results in BMDMs. The cytokine analysis supports the hypothesis generated from the differential gene expression analysis that Tax1bp1-deficiency regulates cytokine responses in AMs. We are very much interested in determining whether these differential cytokine or eicosanoid responses explain the cell type-specific growth of *Mtb*. However, we believe this is beyond the current scope of the manuscript.

(10) LC3 puncta is not a marker of autophagosome maturation. Does Tax1bp1 affect autophagosome formation? The authors at least need to provide western blot for both AM and BMDM.

Good suggestion! We've performed a western blot for LC3 and show the results in Fig. 4-S2. Tax1bp1-deficiency reduced autophagy flux in macrophages, which is consistent with a previous report in HeLa cells (PMID: 23023224).

(11) Does tax1bp1 KO affect cell death without Mtb infection?

Good question. We examined cell death under control conditions – i.e., in the absence of *Mtb* infection – and found that IFN-γ-stimulation of AMs induces apoptosis even in the absence of infection in cells lacking Tax1bp1 (see Fig. 8-S3). These results are consistent with a previous report that Tax1bp1 restrains apoptosis in response to cytokine (TNF-α) stimulation (https://doi.org/10.1038/sj.onc.1202787). Notably, the other cell death phenotypes we discovered (reduced necrotic-like cell death in Tax1bp1-deficient AMs, accelerated apoptosis in IFN-γ-stimulated Tax1bp1-deficient BMDMs) were only observed in the infected cells, not in the uninfected controls.

(12) Autophagy is required for controlling Mtb growth in vivo and ex vivo is less accurate as bactericidal activity of autophagy is still under debate (two important reference missed in the manuscript. PMID: 38413834, 39242815). Please at least rephrase as "Autophagy is required for controlling Mtb in vivo and ex vivo".

Thank you for this point. The manuscript sentence has been revised and includes additional references: "These results contrast with the autophagy receptor function of Tax1bp1 since Tax1bp1 contributes to targeting *Mtb* to selective autophagy (46), a host response that can contribute to the control of Mtb growth in vivo and ex vivo to a variable degree (24,48–55)".

(13) It is very annoying to read when the authors interpreting the data as the function of tax1bp1 rather than stating the phenotype of tax1bp1 deficiency in most places. Such statements also imply for gain-of-function studies, rather than genetic LOF, which deviate from the facts. I agree with reviewer 3 and suggest rewording.

We have modified the entire manuscript, including the title, to focus on the phenotypes associated with the absence of Tax1bp1. In addition, the model now shows the effects of Tax1bp1-deficiency (see Fig. 8E) rather than the function of Tax1bp1.

(14) Line 164 grammar error: Therefore, we measured interferon levels using a type I and II IFN reporter cell line (ISRE) and a type II IFN by ELISA.

This has been corrected.

Flow cytometry files and histopathology slides are included in the Dryad submission along with the microscopy images. The data can be viewed with the following link: http://datadryad.org/share/1JUxAHYmCRwaE5WHXvlJdy0yvV8Bhavl47WTfFEZfP0.

Proteomics data files are available on the ProteomeXchange with identifier PXD064244. Token: XzSBfK5unxae. Alternatively, the dataset can be accessed by logging in to the PRIDE website using the following account details: Username: reviewer_pxd064244@ebi.ac.uk Password: i1yZcJtdnfaO. The complete list of all the changes in protein abundance during *Mtb* infection of alveolar macrophages may be accessed on Dryad.

We appreciate your consideration of this revised manuscript; we believe the new findings, graphical modifications, and major edits to the text have improved it significantly.

Sincerely,

Jonathan Budzik, MD, PhD
Assistant Professor
University of California, San Francisco
