# Supplementary material for: Mycobacterium tuberculosis triggers reduced inflammatory cytokine responses and virulence in mice lacking Tax1bp1
Source: PLoS Pathog. 2025 Oct 31;21(10):e1012829. doi: 10.1371/journal.ppat.1012829 (PMC12588459; doi:10.1371/journal.ppat.1012829)
Supplement: Attachment — Submitted filename: 20250530 Response to Reviewers.docx [file ppat.1012829.s019.docx]

May 30, 2025

Dear Reviewers:

Thank you for reviewing our original research article entitled “Tax1bp1 enhances bacterial virulence and promotes inflammatory responses during Mycobacterium tuberculosis infection of alveolar macrophages” for consideration by PLoS Pathogens. We are grateful for the suggestions for improvement. In order to provide a timely revision of the manuscript, we needed to prioritize our experiments as highlighted in the point-by-point response to the reviewers’ comments and concerns.

**Comments from Reviewer 1**

**Comment 1**: *Experiments are sometimes performed with different readouts (e.g., in Figure 4, CFU for Erdman and luciferase for H37Rv). Another example is mouse kinetics.*

**Response**: To address this concern, we performed both luminescence and CFU measurements in the same experiment in sorted AMs (Figure 4-figure supplement 1). We also performed additional animal experiments at the additional time points (Figure 1-figure supplement 5, Figure 3-figure supplement 2).

**Comment 2**: *The sex of the animals is reported in Figure 1A but not mentioned elsewhere. Is there a sex effect on the susceptibility results in mice? Can you pool male and female results across all in vivo experiments and show sex differences for each group? Along the same lines, could you specify the sex of the mice from which AMs and BMDMs were extracted?*

**Response**: Tax1bp1 enhanced *Mtb* growth in sex-matched mice of both sexes. BAL cells and BMDMs were pooled from mice of both sexes. To address this concern, in the figure legends, we have now specified the sex of the mice used for animal infections (page 20, lines 516-518; page 21, line 543; Supporting information legend Figure 1-figure supplement 1 legend, Figure 1-figure supplement 5, Figure 3-figure supplement 2).

**Comment 3**: *In Figure 1, a survival curve is shown. Would it be possible to include the corresponding weight curve?*

**Response**: The weight data is now presented in Figure 1F.

**Comment 4**: *Furthermore, I do not understand why CFU results at 50 dpi are presented in Figure 1B when WT mice appear to have succumbed to the infection by this time (Fig 1E).*

**Response**: We apologize for the confusion. We note that the time unit for the x-axis in Fig. 1E is weeks and in Fig. 1B is days.

**Comment 5**: *Given the differences in cytokine production and animal survival, it is surprising to observe no changes in cellular infiltration. You have used histology to quantify this, but this technique only detects very significant changes. Could you refine this analysis using flow cytometry to quantify cellular infiltration (e.g., neutrophils) in the lungs? A deeper analysis of the results presented in Supplemental Figure 3.1 might address this question.*

**Response**: We agree. We present this analysis in Figure 1-figure supplement 5.

**Comment 6**: *Is it possible to show the weight and survival curves for the Listeria infection model? I also suggest emphasizing the relevance of testing this model in your study.*

**Response**: We unfortunately did not collect weight data in our original experiments with *Listeria* and did not carry out infections with later time points. The readouts used in our study (CFU and cytokine levels) are typically used in the field. PMID 22393157 and 20956571 are examples.

**Comment 7**: *It is known that Tax1bp1 is involved in the autophagy process. However, the colocalization results of LC3 observed very early after Mtb infection suggest that early vacuole maturation could be affected. Is it possible to verify its integrity (phagosomal rupture?) and also track the acidification of the Mtb-containing vacuole in WT and KO macrophages (in line with the results of Figure 5)?*

**Response**: We agree that these experiments would be very interesting to perform. We followed the editor’s suggestion of focusing on additional animal experiments for this revised manuscript.

**Comment 8**: *Regarding the reversed phenotype observed between AMs and BMDMs, is it possible to determine the expression level of Tax1bp1 over time? This could provide insights into how Mtb manipulates it.*

**Response**: We are grateful for the reviewer’s suggestion to measure Tax1bp1 protein levels over time. This data is now presented in Figure 5 and described on pages 12-13, lines 297-337. Interestingly, Tax1bp1 protein level did not change in *Mtb-*infected AMs compared to mock-infected AMs. However, there was a significant increase in Tax1bp1 during *Mtb* infection of BMDMs. We agree with the reviewer that this is an important insight into how *Mtb* manipulates Tax1bp1 in different cells.

**Comment 9**: *Additionally, could you reproduce experiments with one of the Mtb mutants identified in the dual RNAseq analysis?*

**Response**: We agree that this would be a very interesting follow-up experiment to perform. However, generating *Mtb* mutants is time-consuming, and we believe the suggested experiment is outside the scope of the current study.

**Comment 10**: *Concerning the results showing that WT macrophages undergo more necrosis than KO macrophages, is it possible to demonstrate this in the lungs of infected mice? Could you add a specific staining to your histological sections and quantify it? Alternatively, could flow cytometry be used to assess this?*

**Response**: We agree that it is important to test whether the findings from the *ex vivo* AM infection model also occur *in vivo*. By gating for live single cells together with quantification of recruited immune cells, we now show in Figure 1-supplement 5 that Tax1bp1 leads to a decreased proportion of AMs at day 28, which may suggest increased AM cell death driven by Tax1bp1 (page 7, lines 180-183).

**Comments 11 and 12**: *To confirm the hypothesis proposed in line 370, could you compare Mtb behavior in bone marrow-derived cells cultured with MCSF (BMDM) or GMSCF (BMDC)? If the conclusion is that Tax1bp1 facilitates infection in AMs but restricts it in BMDMs, could you test WT and KO mice with aerosol versus intraperitoneal (IP) infection? In this case, you might observe greater susceptibility in the lungs of WT mice following aerosol infection (as shown in Figure 1) and potentially greater resistance in the spleen and liver following IV infection.*

**Response**: While we agree that these additional experiments may inform the role of Tax1bp1 in non-physiological routes of *Mtb* infection, given the very extensive additional work required, we believe these to be beyond the scope of the current study. We do not think lack of these data invalidates our proposed hypothesis, which is demonstrated by our *ex-vivo* experiments with AMs vs. BMDMs.

**Comments from Reviewer 2**

**Comment 1**: *In the first paragraph, the authors state: "Our results suggest that Tax1bp1 amplifies host-detrimental inflammatory responses, which predominate over cell-intrinsic control of Mtb replication by autophagy in macrophages (48) and contribute to host susceptibility and mortality." However, this conclusion is not supported by the data presented. The paragraph does not include specific analyses of macrophages, and the authors themselves note: "Although Tax1bp1 contributes to inflammatory cytokine synthesis during Mtb infection, microscopic examination of infected lung tissue did not reveal any significant differences in the cellular infiltrate of the lungs as reflected by lesion severity or tissue necrosis." This statement directly contradicts their assertion that host-detrimental inflammatory processes dominate over autophagy.*

**Response**: We agree that this statement may seem contradictory. However, as suggested by the first reviewer, histologic assessment of inflammation may be less sensitive than flow cytometry at detecting changes in immune cell recruitment. Indeed, following flow cytometry analysis, we now show that there are important differences in immune cell recruitment mediated by Tax1bp1 (Figure 1-figure supplement 5). This section of the manuscript has been edited accordingly (pages 7-8, lines 173-192).

**Comment 2**: *Based on the data presented, the observed differences in cytokine levels are more likely due to systemic inflammatory regulation rather than localized macrophage dysfunction or tissue damage. Furthermore, the statistically significant differences in cytokine levels are observed only at 21 and 50 days post-infection, corresponding to the time when monocyte-derived cells migrate to the lung parenchyma and become infected (as described in studies by Cohen et al. and Huang et al.). This strongly supports the idea that the inflammatory regulation is systemic in nature.*

*To strengthen the study, the authors should provide an analysis of the proportions of CD4/CD8 T-cells between WT and KO mice at early and late time points. This is especially important given that this is a whole-animal knockout model, not a myeloid-specific knockout. The deletion of Tax1bp1 is therefore likely to have broader impacts on T-cell function and could be a key factor influencing the observed cytokine differences.*

**Response**: Thank you for this suggestion. We appreciate the reviewer’s comment and performed the analysis of T cell recruitment at day 14, 21, and 28 time points (Figure 1-figure supplement 5; pages 7-8, lines 175-192). The new results show that Tax1bp1 enhances neutrophil and CD8+ T cell recruitment and support the idea that Tax1bp1 regulates systemic inflammatory responses. I changed the title of the manuscript to be less focused on the impact of Tax1bp1 on inflammatory responses in AMs.

**Comment 3**: *The transcriptional analysis presented in the manuscript raises significant concerns. First, how do the authors reconcile their earlier finding that Tax1bp1 promotes Mtb growth in alveolar macrophages (AMs) with the observation that there are no significant transcriptional differences in Mtb between Tax1bp1 WT and KO AMs? If Mtb growth is enhanced in WT AMs but restricted in KO AMs, one would expect transcriptional changes in the bacteria reflecting this difference in growth conditions. The absence of such differences is a critical inconsistency, raising the possibility that the observed host effects are driven by systemic immune responses rather than the direct function of Tax1bp1 in AMs. The statement, "These results are consistent with our observation that Tax1bp1 enhances increased intracellular Mtb growth during AM infection (Figure 4)," should be removed. The authors selectively highlight a few bacterial genes whose expression differences between WT and KO AMs are not statistically significant, yet claim these results support their conclusions. Such cherry-picking is misleading and does not substantiate their argument.*

**Response**: One possible explanation for this result is that our transcriptional profiling was performed before significant changes in *Mtb* growth were observed beginning at 48 hours post-infection (Figure 4). A possible explanation for the lack of dramatic *Mtb* transcriptional changes is that Tax1bp1 impacts the killing (or restriction) of *Mtb,* as opposed to leading to changes in *Mtb* replication. The former might not produce transcriptional changes in *Mtb,* whereas the latter would. Based on the reviewer’s comment, please note that we have edited the conclusions in this section of the manuscript (page 11, lines 348-354) and removed the sentence in quotations.

**Comment 4**: *Regarding the host transcriptional analysis, the authors list several genes (Cd4, Cxcl1, Pf4, Pdgfa, Kitl, and Cxcl3) that they speculate are correlated with increased necrosis in WT AMs. However, no references or evidence are provided to support these claims. Moreover, the authors suggest that necrosis in WT AMs results from pro-inflammatory responses, yet their data in Figure 6 show that the intrinsic apoptotic pathway is the most upregulated in WT AMs. Adding to the confusion, they also state that pro-apoptotic genes are among the top upregulated genes in KO AMs. This contradiction undermines their conclusion that Tax1bp1 differentially regulates necrotic and apoptotic pathways.*

**Response**: Thank you for pointing out the confusing aspects of this section. We edited the manuscript to denote that the genes listed are members of specific Gene Ontogeny pathways and provided references for each gene. We wish to point out that the gene ontogeny enrichment analysis was performed on genes that were both upregulated and downregulated. Given that the effect of individual inflammatory and cell death genes can be difficult to predict given the complexity of their signalling pathways, we measured the cytokines and host cell death in *Mtb*-infected cells to follow up on the hypotheses generated by the RNAseq analysis. Accordingly, we have edited this section of the manuscript (pages 13-14, lines 339-376).

**Comment 5***: In the Discussion section, the authors claim that "Tax1bp1 controls the mode of host cell death by initially promoting necrotic-like cell death in the first four days of Mtb infection in AMs but not BMDMs, while delaying apoptosis in the later stages of infection." However, the presented data do not support this statement and fail to demonstrate clear differences in cell death modes over time.*

**Response**: To improve the clarity of the section, in the revised manuscript, we point out the specific time points during which there were statistically significant differences in the levels of fluorescent-stained cells (page 11, lines 413-418). We removed the data from day 8 because the macrophages all died by day 7. This will help the reader focus on the relevant time points for these experiments.

**Comment 6**: *Finally, the GEO repository containing the transcriptional data is locked until 2028, and no password has been provided for access. This prevents reviewers from verifying the analysis. To ensure transparency and reproducibility, the authors must provide processed data files (e.g., gene count matrices) and the code used for the analysis (e.g., R scripts), allowing reviewers to validate their findings.*

**Response**: We apologize for this oversight. The code for accessing the analysis was included in our original cover letter with the initial submission of the manuscript. The following private access token can be used to access the RNAseq sequencing files and differential gene expression analysis deposited in GEO: svczakgyvvuldoz. The link is: <https://www.ncbi.nlm.nih.gov/geo/query/acc.cgi?acc=GSE280399>.

**Comment 7**: *In the paragraph titled "Tax1bp1 promotes inflammatory cytokine signaling during AM infection ex vivo," how do the authors reconcile their findings with previous studies, such as the work by Rotchild et al., which demonstrated that AMs are unable to mount a pro-inflammatory response during the first 10 days of Mtb infection? This discrepancy is not addressed in the manuscript.*

**Response**: Thank you for pointing out this distinction between the results performed by Rothchild *et al. in vivo* and our responses in AMs infected *ex vivo.* We believe there are factors present *in vivo* that may influence the ability of AMs to mount pro-inflammatory responses in the lung environment. Please note that we now address this point in the Discussion section of the revised manuscript (page 17, lines 436-446).

**Comment 8**: *Additionally, it is not clear from the text or the Materials and Methods section at what time point the AMs were isolated. Were they harvested from naive mice, or from infected mice? If the latter, what was the time point post-infection? This information is critical for interpreting the observed inflammatory responses.*

**Response**: Thank you for highlighting an important omission. AMs were harvested from naïve mice, which we have now specified in the methods.

**Comment 9**: *Another methodological concern is that AMs were isolated via bronchoalveolar lavage (BAL) without any further purification. It is well-established that BAL contains a heterogeneous population of cells, including tissue-resident alveolar macrophages, monocyte-derived alveolar macrophages (MoAMs), T-cells, and epithelial cells. Recent work by Russell and colleagues has shown that MoAMs are highly pro-inflammatory upon Mtb infection, in contrast to tissue-resident macrophages, which are unable to mount such responses. Thus, the observed inflammatory responses are likely driven by MoAMs, which behave similarly to bone marrow-derived macrophages (BMDMs).*

**Response**: We appreciate that *Mtb* infection causes the heterogeneity of immune cells to increase significantly. However, our experiments were performed with BAL cells from naïve mice, which do not have MoAMs in the lungs because MoAMs are recruited after *Mtb* infection. Therefore, our infection experiments with AMs *ex vivo* are not impacted by these specific cells, although there may be other contaminating cells in the BAL fluid. We edited the discussion accordingly (page 18, lines 475-483).

**Comment 10**: *To address this issue, the authors should repeat these experiments using SiglecF-based purification to isolate tissue-resident AMs, excluding MoAMs and other contaminating cell types such as T-cells. This refinement is particularly important for subsequent necrotic and apoptotic readouts, as these responses may also be confounded by the inclusion of MoAMs and other cell populations.*

**Response**: We agree with the suggestion to perform experiments in purified AMs from the BAL. We show new data using sorted AMs in Figure 4-figure supplement 1. The conclusion from this experiment is that Tax1bp1 supports *Mtb* growth in sorted AMs, like it does in the BAL cells (page 11, lines 275-278). We wished to perform additional infections *ex vivo* with sorted AMs, but we were limited by the size of our mouse colony.

**Comment 11**: *However, the most important issue of this study, which leads me to recommend rejection of the manuscript, is the lack of standardization and consistency in the ex vivo and in vitro experiments. According to the Materials and Methods section, infections of AMs were performed using a range of MOIs: MOI 1 in some experiments, MOI 2 in others, and MOI 10 in yet others.*

*Infection of macrophages at varying MOIs induces different levels of host cell activation and results in distinct readouts. For instance, infecting a monolayer at MOI 1 is fundamentally different in its cellular and immunological impact compared to MOI 10, generating data that are not directly comparable.*

*The majority of the results in this manuscript rely on ex vivo infections of AM monolayers, and these experiments are subsequently used to build a narrative and support the overarching conclusions. However, the inconsistent infection conditions raise concerns about the scientific validity of the study. This variability gives the impression that the experimental parameters may have been adjusted to align with the desired narrative, which undermines confidence in the findings.*

*For this study to be scientifically robust and relevant, the infection conditions should be standardized. All experiments should utilize consistent infection protocols, ensuring that the results are comparable and provide a reliable basis for the conclusions drawn.*

**Response**: Thank you for this comment and suggestion. Regarding the concern that the MOI changed between experiments, various MOIs were selected for two reasons: (1) for comparison of our results in AMs with our previously published results in BMDMs, and (2) for obtaining different readouts, as is standard in the field. For example, we performed the *Mtb* growth assay at a low MOI of 1 to allow the *Mtb* growth assay to proceed for 4 days. Because macrophages burst with an intracellular *Mtb* load of 25 (PMID: 23436998), *Mtb* growth assays at a high MOI must be carried out over a short time frame, during which *Mtb* has less of an opportunity to grow. On the other hand, we performed the cytokine measurements from the infected cell supernatants at a higher MOI of 5 and a shorter time point of 24 hours because cytokine responses to *Mtb* are extremely low at an MOI of 1 (PMID: 37963018). A disadvantage of the MOI of 1 is that one study showed that only about 80% of cells are infected with *Mtb* at a MOI of 1 (PMID: 31636345). Because we wished to increase the likelihood of all host cells being infected with *Mtb*, we performed immune fluorescence microscopy experiments at a MOI of 2, which is also consistent with our previously published experiments in BMDMs.

In the revised manuscript, I describe the rationale behind using a higher MOI for cytokine assays (page 15, lines 385-387) and proteomic analysis (page 12, lines 307-310).

**Comments from Reviewer 3**

**Comment 1**: *Despite efforts made using both relevant in vivo and ex-vivo experiments, I am concerned about the number of biological replicates provided in this manuscript. At this stage of submission, many of the experiments are only performed once or twice by biological replicate, especially key experiments supporting the conclusions. In Figure 1 and Supplemental Figure 1 describes two similar experiments done in 5 mice each time, but in a single set of experiments. An additional third experiment should be important to provide clear evidence about the role of TAX1BP1 in vivo.*

**Response**: Thank you for this suggestion. For clarification, Figure 1 was an independent experiment from Figure 1-figure 1. Regarding the concern that only two replicate experiments (Figure 1 and Supplemental Figure 1) were performed, a third *in vivo* experiment was performed (Figure 3A), demonstrating that Tax1bp1 enhances *Mtb* growth *in vivo*.

We did not have enough mice in our colony to perform three replicates of every experiment. We made additional attempts at repeating the *Mtb* growth assay in the AMs expressing phosphomutant Tax1bp1 alleles. However, since additional attempts at regenerating the AMs expressing Tax1bp1 phosphomutant alleles were not successful due to an unclear issue with lentiviral transduction of the primary AMs, we could not perform replicates for the original experiment, and therefore, we removed the data from the manuscript.

Additional replicate experiments were performed for the cell death assay in AMs infected *ex vivo*, which we have now indicated in the figure legend for Figure 8 (page 23, lines 589-590), Figure 8-figure supplement 1 (Supporting Information page 3), and Figure 8-figure supplement 2 (Supporting Information page 3). An additional replicate experiment was performed for the autophagy microscopy experiments as indicated in the legend of Figure 4-figure supplement 2 (Supporting Information page 4).

An important conclusion of this manuscript is that Tax1bp1 has a cell type-specific impact on *Mtb.* Accordingly, this revision now has four independent animal infection experiments testing this hypothesis (Figure 1-figure supplement 5, Figure 3, Figure 3-figure supplement 1, and Figure 3-figure supplement 2).

**Comment 2**: *In Figure 4, AMs isolation in BALs is described in the material and methods section. However, the authors need to provide characterization evidence that those cells are indeed AMs (flow cytometry, immunofluorescence for example). This characterisation is important for the conclusions.*

**Response**: Thank you for this suggestion. In the revised manuscript, we characterized the BAL cells by flow cytometry (Figure 4-figure supplement 1). This revealed that 96.8% and 95.6% of live single cells in the BAL fluid are Siglec-F^+^Cd11c^+^ AMs from wild-type and *Tax1bp1^-/-^* mice, respectively (page 11, lines 278-279).

**Comment 3**: *How these cells compared with the Siglec+ and Siglec- cells from Pisu et al. 2021?*

**Response**: In response to this question, we include in the discussion section that Siglec- AMs are found in the lungs after *Mtb* infection (page 18, lines 475-483). Because we performed BAL in naïve mice, Siglec- AMs are not present in our *ex vivo* infection experiments with AMs.

**Comment 4**: *Related to the figure 5: On one hand, LC3 puncta, as reviewed in Kumar et al. 2021, should not be considered as a marker of autophagosomes. To confirm that those are autophagosomes, the authors should either provide TEM evidence or genetic evidence (ATG7 vs ATG13 KO for example). On the other hand, autophagosome maturation, which is the fusion of those structures with lysosomal compartments is not shown. Ideally, the authors could use the tandem RFP-GFP LC3 construct to provide such information. Alternatively, the authors could include this figure in the supplemental section and describe that they observed similar phenotypes between BMDMs and AMs leading them to further characterize this role of TAX1BP1 with different approaches.*

**Response**: Thank you for pointing this out. We edited this section of the manuscript (pages 11-12, lines 287-296). Due to limitations in the size of our mouse colony, we focused on performing additional animal experiments to strengthen this manuscript revision.

**Comment 5**: *There is an important connection here between nitric oxide, cell death and macrophage control of Mtb. It will be important here to determine NO release from both BMDM and AM to define if autophagy independent, NO-dependent mechanisms are the underlying reason for the observed differences.*

**Response**: We agree that this is an important experiment to perform to further probe the autophagy independent mechanisms of Tax1bp1 in the control of *Mtb.* As mentioned previously, we focused on performing additional *in vivo* experiments for this manuscript revision.

**Comment 6**:  *In the entire manuscript, individual values should be plotted in bar charts.*

**Response**: Thank you for this suggestion. The graphs have been replotted.

**Comment 7:** *In the main text, the authors systematically write the effect of tax1bp1 KO as “TAX1BP1 is acting… “. The available data, which are comparing wt and tax1bp1 KO mice cannot provide evidence, unless the authors find a model where they rescue tax1bp1 expression. The authors should reword their findings such as “tax1bp1 KO led to decreased Mtb replication suggesting that TAX1BP1 plays a pro-bacterial role”*

**Response**: We appreciate this suggestion. However, we believe that the manuscript is easier to read and conceptualize when phrased to point out the effects of Tax1bp1, as opposed to the effects of the lack of Tax1bp1. In this revision, we have added a paragraph in the discussion section noting the limitation of our use of the Tax1bp1-deficient mouse model (page 19, lines 494-501).

**Comment 8**: *In Figure 1, the authors should harmonize their representation as they separate male and female mice only in the first timepoint, without providing explanation in the text. Interestingly, it seems that the uptake of Mtb between male and female in tax1bp1 KO is different. Could the authors comment on this?*

**Response**: Thank you for this comment. It is true that there is a difference in uptake between male and female Tax1bp1-deficient mice. We revised the manuscript accordingly (page 6, lines 147-149). To control for sex as a biological variable, at each time point, sex-matched mice were harvested. As we have now noted in the figure legends, at each time point and in each *Mtb* animal experiment, the sex is specified.

**Comment 9**: *Figure 1 Supplemental 1A, it could be a coincidence, but the p-values at 21 and 50 days are similar to those shown in Fig1B. Could the authors double-check to make sure if those values were not duplicated?*

**Response**: Thank you for highlighting this. We double-checked the p values.

**Comment 10**: *Figure1 Supplemental 3, the authors could include a sentence about the ubiquitin signal increase shown in tax1bp1 KO as it is something shown in the brain (Sarraf et al. 2020).*

**Response**: Thank you for this suggestion. The section of the text describing Figure 1-figure supplement 3 has been modified accordingly (page 8, line 206). The discussion has also been edited to incorporate key findings from this manuscript (page 19, line 495-498).

**Comment 11**: *Figure 3 Supplemental 2, It is unclear why the authors did not provide the CFU count for this experiment.*

**Response**: We attempted to measure the CFU count for the experiment in Figure 3 Supplemental 2. However, colonies did not grow due to an error in plate preparation. For this reason, we only have flow cytometry analysis from this experiment.

**Comment 12**: *In Figure 5, the conclusion of this figure is not in accordance with the presented data. LC3 puncta colocalization with Mtb (as well as ubiquitin) is not a marker of autophagosome maturation. The quality of the imaging is poor, and it is unclear how the quantifications were done as the images are of poor quality.*

**Response**: Thank you for the comment. Quantification was performed by enumerating the percentage of *Mtb* that overlapped with the autophagy marker (ubiquitin or LC3). We edited this section of the manuscript based on the reviewer’s comments (pages 11-12, lines 287-296). The images we obtained are typical of those obtained on high-content imaging micrographs that are used for quantification. In this manuscript, we used the same brand of microscope and image analysis software as used for our previous publication (PMID 31951200).

**Comment 13**: *In Figure 7, The data shown in Fig7 highlight a decrease of pro-inflammatory cytokines upon tax1bp1 KO in a timepoint where Mtb replication is not affected. The authors should provide evidence, in later timepoint and in the same experiment, that tax1bp1 KO decrease Mtb replication consistent with their previous findings.*

**Response**: Thank you for this suggestion. We prioritized performing additional animal experiments for the manuscript revision.

**Comment 14**: In Figure 8, the images provided show clearly that the number of cells is not homogeneous. Hence, to be more consistent, the quantification of PI+ cells and CellEvent+ cells should be normalized by the total number of cells (quantifiable using the brightfield channel).

**Response**: Thank you for this suggestion. In the revised manuscript, the microscopy data have been normalized to the total number of cells in the phase contrast channel.

**Comment 15**: The authors should also provide a control without infection to ensure that tax1bp1 KO does not affect cell death in their conditions.

**Response:** We appreciate the rationale behind providing this control. Because this was raised in the Minor Issue section, we prioritized using the available mice in our colony to address the Major Issues by performing additional *in vivo* infection experiments.

Flow cytometry files and histopathology slides are now included in the Dryad submission along with the microscopy images. The data can be viewed with the following link: [http://datadryad.org/share/1JUxAHYmCRwaE5WHXvlJdy0yvV8Bhavl47WTfFEZfP0](https://urldefense.com/v3/__http:/h52wvrds.r.us-west-2.awstrack.me/L0/http:*2F*2Fdatadryad.org*2Fshare*2F1JUxAHYmCRwaE5WHXvlJdy0yvV8Bhavl47WTfFEZfP0/1/01010196ee994d09-10ab2212-2330-4dbf-82e2-b7a8914cbbeb-000000/ctBzV4O-XY0W8BBk5s4z7bC6hOE=427__;JSUlJQ!!LQC6Cpwp!uyUTdeBUElr2fr_9WC6HTUXCPWQgOplPY9tTW0Jb9J4ZuhgIdWVS0hdn9B46OG_6jn5xqQjQ-55uF-ruNvCe4jHycsBIi7fX-4vr0w$).

Proteomics data files are available on the ProteomeXchange with identifier PXD064244. Token: XzSBfK5unxae. Alternatively, the dataset can be accessed by logging in to the PRIDE website using the following account details: Username: [reviewer_pxd064244@ebi.ac.uk](mailto:reviewer_pxd064244@ebi.ac.uk) Password: i1yZcJtdnfaO. The complete list of all the changes in protein abundance during *Mtb* infection of alveolar macrophages may be accessed on Dryad.

We believe the new findings from the additional experiments presented in this revision and the major edits to the text have made significant improvements to the manuscript.

I appreciate your consideration.

Sincerely,

Jonathan Budzik, MD, PhD

Assistant Professor of Medicine

University of California, San Francisco
